# LRPPRC and SLIRP synergize to maintain sufficient and orderly mammalian mitochondrial translation

**DOI:** 10.1093/nar/gkae662

**Published:** 2024-08-01

**Authors:** Diana Rubalcava-Gracia, Kristina Bubb, Fredrik Levander, Stephen P Burr, Amelie V August, Patrick F Chinnery, Camilla Koolmeister, Nils-Göran Larsson

**Affiliations:** Division of Molecular Metabolism, Department of Medical Biochemistry and Biophysics, Karolinska Institutet, Stockholm, Sweden; Division of Molecular Metabolism, Department of Medical Biochemistry and Biophysics, Karolinska Institutet, Stockholm, Sweden; Department en Immunotechnology, National Bioinformatics Infrastructure Sweden, Science for Life Laboratory, Lund University, Lund, Sweden; Department of Clinical Neurosciences, School of Clinical Medicine, University of Cambridge, Cambridge Biomedical Campus, Cambridge, UK; Medical Research Council Mitochondrial Biology Unit,University of Cambridge, Cambridge Biomedical Campus, Cambridge, UK; Division of Molecular Metabolism, Department of Medical Biochemistry and Biophysics, Karolinska Institutet, Stockholm, Sweden; Department of Clinical Neurosciences, School of Clinical Medicine, University of Cambridge, Cambridge Biomedical Campus, Cambridge, UK; Medical Research Council Mitochondrial Biology Unit,University of Cambridge, Cambridge Biomedical Campus, Cambridge, UK; Division of Molecular Metabolism, Department of Medical Biochemistry and Biophysics, Karolinska Institutet, Stockholm, Sweden; Division of Molecular Metabolism, Department of Medical Biochemistry and Biophysics, Karolinska Institutet, Stockholm, Sweden

## Abstract

In mammals, the leucine-rich pentatricopeptide repeat protein (LRPPRC) and the stem-loop interacting RNA-binding protein (SLIRP) form a complex in the mitochondrial matrix that is required throughout the life cycle of most mitochondrial mRNAs. Although pathogenic mutations in the *LRPPRC* and *SLIRP* genes cause devastating human mitochondrial diseases, the *in vivo* function of the corresponding proteins is incompletely understood. We show here that loss of SLIRP in mice causes a decrease of complex I levels whereas other OXPHOS complexes are unaffected. We generated knock-in mice to study the *in vivo* interdependency of SLIRP and LRPPRC by mutating specific amino acids necessary for protein complex formation. When protein complex formation is disrupted, LRPPRC is partially degraded and SLIRP disappears. Livers from *Lrpprc* knock-in mice had impaired mitochondrial translation except for a marked increase in the synthesis of ATP8. Furthermore, the introduction of a heteroplasmic pathogenic mtDNA mutation (m.C5024T of the tRNA^Ala^ gene) into *Slirp* knockout mice causes an additive effect on mitochondrial translation leading to embryonic lethality and reduced growth of mouse embryonic fibroblasts. To summarize, we report that the LRPPRC/SLIRP protein complex is critical for maintaining normal complex I levels and that it also coordinates mitochondrial translation in a tissue-specific manner.

## Graphical Abstract

**Graphical Abstract:**
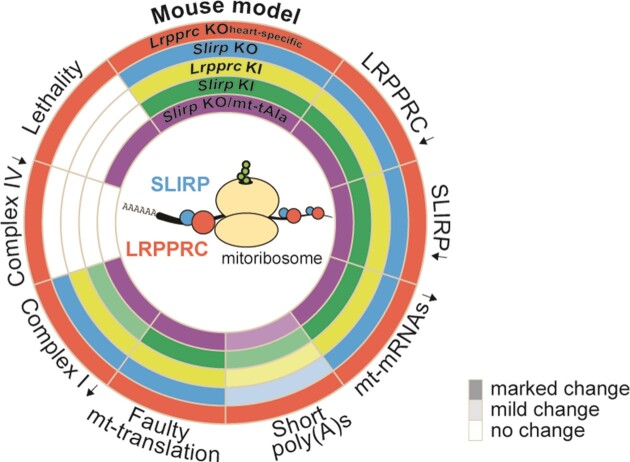

## Introduction

Mitochondria execute a plethora of metabolic functions, including the conversion of harvested energy into adenosine triphosphate (ATP) via oxidative phosphorylation (OXPHOS) (1). Most eukaryotic cells contain mitochondrial DNA (mtDNA), which is present in multiple copies per cell and distributed along the mitochondrial network (2,3). In mammals, mtDNA may be present in identical copies (homoplasmy) or vary in sequence in a proportion of the molecules (heteroplasmy). Metabolic defects manifest when a pathogenic heteroplasmic variant exceeds a critical threshold and OXPHOS function is impaired (4). Mammalian mtDNA encodes two ribosomal RNAs (12S and 16S mt-rRNAs), the complete set of 22 transfer RNAs (mt-tRNAs) required for translation, and 11 messenger RNAs (mt-mRNAs), including two bicistrons, that are translated into 13 proteins (5). Each strand of mtDNA (the light and heavy strand) is transcribed into a long primary polycistron that is rapidly processed co-transcriptionally to release the individual transcripts (6,7). Each mtDNA-encoded protein assembles into its corresponding OXPHOS complex along with nucleus-encoded subunits. For instance, the mammalian OXPHOS complex I is composed of 45 subunits counting seven that are mtDNA-encoded. Similarly, complex III, IV and the ATP synthetase (complex V) are composed of subunits derived from two genetic origins (8). The rest of the hundreds of proteins required to fulfill mitochondrial functions, and for mtDNA gene expression, are synthesized in the cytosol and imported to mitochondria (9).

The leucine-rich pentatricopeptide repeat protein (LRPPRC) in complex with the stem loop interacting RNA-binding protein (SLIRP) binds and stabilizes most mt-mRNAs co-transcriptionally by hindering the activity of the PNPase exonuclease and by promoting the polyadenylation of mt-mRNA by the mitochondrial poly-A polymerase (MTPAP) (10–13). Moreover, the LRPPRC/SLIRP complex is required for proper loading of mt-mRNAs into mitochondrial ribosomes (mitoribosomes) (11,14). A notable exception is the *ND6* mt-mRNA, the only coding light-strand transcript, which is neither bound by the LRPPRC/SLIRP complex nor polyadenylated (11,15). LRPPRC contains 33 pentatricopeptide repeat (PPR) domains (15,16). These helix-turn-helix domains in tandem form a superhelix groove that binds and stabilizes RNA (17–19). SLIRP is much smaller than LRPPRC and contains an RNA recognition motif, which, at least *in vitro*, does not interact with mt-mRNAs (20).

Mutations in human *LRPPRC* cause the French-Canadian type of Leigh Syndrome, characterized by an infantile onset of severe neurodegeneration and a deficiency of complex IV (21,22). The recently described pathogenic variants in *SLIRP* cause mitochondrial encephalomyopathy with complex I and complex IV deficiency (23). The lack of either LRPPRC or SLIRP in mice results in markedly decreased mt-mRNA levels (11,14). However, the phenotypes of these two knockout models are highly contrasting. The full-body knockout of *Lrpprc* (*Lrpprc*^−/−^) causes embryonic lethality, and the heart- and muscle-specific knockouts do not survive past 14 weeks of age. Also, mitochondrial translation becomes chaotic in *Lrpprc*^−/−^ hearts with some peptides being overproduced and others less synthesized (11). In contrast, *Slirp* full-body knockout (*Slirp*^−/−^) mice are essentially healthy despite an overall decrease in mitochondrial translation in some tissues (14).

SLIRP becomes undetectable in the absence of LRPPRC, inversely, LRPPRC levels drop down to ∼25% in the absence of SLIRP. This interdependence in stability has restrained the study of any individual functions for these proteins. Also, the defect in mitochondrial translation caused by the lack of SLIRP is tissue dependent. Further, determining the complete 3D structure of LRPPRC has thus far escaped all attempts, a partial structure together with SLIRP has been recently resolved in association with the mitoribosome (24). In this structure, SLIRP and LRPPRC contact the mt-mRNA, and LRPPRC associates with the mitoribosome to deliver the mRNA for translation. As the LRPPRC/SLIRP complex acts as a global chaperone throughout the RNA-related steps of mtDNA gene expression, it seems unlikely that a single structural model and interaction pattern represents all roles fulfilled by this complex. In addition, there is an apparent surplus of mt-mRNAs and only a fraction interacts with the mitoribosome, whereas the remaining pool of untranslated mt-mRNAs is bound by LRPPRC/SLIRP. Loss of SLIRP leads to a drastic decrease of the pool of untranslated mt-mRNAs without causing any obvious phenotype in the mouse. However, it is unclear if the pool of untranslated mt-mRNAs is needed when conditions deviate from normal physiology.

In this study, we performed detailed studies of the *in vivo* functions of LRPPRC and SLIRP by employing several mouse models with disrupted or mutated *Lrpprc* and *Slirp* genes. The proteome of *Slirp*^−/−^ mitochondria from heart and liver showed a decrease in complex I subunits. We impeded the interaction between LRPPRC and SLIRP *in vivo* by generating two mouse lines carrying knock-in mutations of either the *Lrpprc* or *Slirp* genes. LRPPRC levels are reduced and SLIRP completely disappears when these proteins do not interact, indicating a tightly controlled turnover of SLIRP. Livers from *Lrpprc* knock-in (*Lrpprc*^ki/ki^) mice showed a general decrease in mitochondrial translation except for a marked increase in the synthesis of ATP8. Interestingly, we detected mitoribosomes with a sedimentation pattern characteristic of polysome formation when SLIRP/LRPPRC levels are low, suggesting that multiple mitoribosomes engage on each mt-transcript when mt-mRNA levels are low, and translation not properly coordinated. Finally, we asked if the defects caused by the absence of SLIRP are aggravated when mitochondrial translation is disrupted by an independent defect. Introducing the heteroplasmic m.C5024T mutation of the tRNA^Ala^ gene of mtDNA into *Slirp*^−/−^ mice leads to an additive defect further reducing mitochondrial translation causing embryonic lethality as well as reduced proliferation of embryonic fibroblasts under oxidative conditions. To summarize, we report that LRPPRC and SLIRP synergize to maintain orderly mtDNA gene expression that affect levels of complex I and mouse embryonic development.

## Materials and methods

### Generation of animal models

All mouse strains used were derived from the C57BL/6N background. The *Slirp* knockout (*Slirp*^−/−^) mouse line, the mouse females with the m.C5024T mutation in the tRNA^Ala^ gene of mtDNA, the tissue-specific *Lrpprc* knockout (*Lrpprc*^−/−^), and the MitoRibo-Tag (*mL62-Flag*^tg/tg^) mice were generated as previously described (11,14,25,26). CRISPR/Cas9 gene editing technology was used to generate the *Lrpprc* (*Lrpprc*^ki/ki^) and *Slirp* knock-in (*Slirp*^ki/ki^) lines. Briefly, pronuclear-state zygotes from C57BL/6N background were injected with sgRNA, recombinant Cas9 protein (Sigma), and ssODN as recombination template, all at 20 ng/μl. Details of all sequences are provided in Supplementary Table S3. Injected zygotes were implanted into pseudo pregnant females to obtain the founder generation offspring. Mice were backcrossed to C57BL/6N mice for four generations before intercrossing each knock-in line.

### Ethics statement

The animal experiments were performed in accordance with the recommendations and guidelines of the Federation of European Laboratory Animal Science Associations (FELASA). Animal studies were approved by the animal welfare ethics committee and performed in compliance with National and European law.

### Mitochondria isolation

Heart, liver and kidney tissues were dissected from CO_2_-euthanized mice and preserved in ice-cold PBS for fresh isolation of mitochondria by differential centrifugation. All steps in this section were performed on ice, using ice-cold solutions and all centrifugations were performed at 4°C. Tissues were cut into pieces and added to Potter-homogenizer tubes containing 5 ml of mitochondria isolation buffer (320 mM sucrose, 10 mM Tris–HCl, pH 7.4, 1 mM EDTA, 0.1% BSA) supplemented with protease inhibitor (Roche). Optionally, heart pieces were rotated for ten minutes in 2 mL of mitochondria isolation buffer without BSA and containing 0.25% of Trypsin (this step improves yield and was used for preparing mass spec samples and for [^35^S]-labelling experiments). Trypsin-treated heart samples were supplemented with 3 mL of mitochondria isolation buffer containing BSA and trypsin inhibitor (0.4 mg/ml). Tissues were homogenized using a Potter-homogenizer on ice (15 strokes, 450 rpm) and nuclei and cell debris were pelleted by spinning at 1000g for 10 min. The supernatant was further centrifuged at 10 000g for 10 min. Pellets were resuspended in mitochondria isolation buffer lacking BSA and centrifuged again at 10 000g for 10 min. Mitochondrial protein concentrations were determined using colorimetric assays (Pierce BCA Protein Assay Kit) according to the manufacturer's instructions.

For obtaining Percoll-purified mitochondria, Percoll gradients were prepared in ultracentrifugation tubes (Beckman Ultraclear 14 × 89 mm) by carefully overlaying 4 mL of mitochondria purification buffer (220 mM mannitol, 70 mM sucrose, 5 mM HEPES–KOH, pH 7.6 and 1 mM EGTA, pH 7.4 supplemented with protease inhibitor (Roche)) containing 19% Percoll on 2 ml of mitochondria purification buffer containing 40% Percoll without disturbing the interphase. Then, freshly isolated mitochondria were resuspended in 4 ml of mitochondria purification buffer containing 12% Percoll and added to the top of the Percoll-gradient. Samples were ultracentrifuged using a SW41 Ti swing-out rotor at 42 000g for 30 min without deceleration. Purified mitochondria were harvested from the interphase layer between 19 and 40% Percoll and washed twice with 10 ml of mitochondria purification buffer. Mitochondrial pellets were snap frozen in liquid nitrogen and stored at −80°C until further use.

### Mitoribosome co-immunoprecipitations

Percoll-purified mitochondria (1 mg) were resuspended in 200 μl of lysis buffer (10 mM Tris–HCl pH 7.5, 100 mM potassium chloride (KCl), 20 mM magnesium dichloride (MgCl_2_), 1× protease inhibitor (Roche), 1% digitonin). The lysates were incubated for 20 min on ice and centrifuged for at 9200g for 45 min at 4°C. The resulting supernatants were diluted 1:10 with dilution buffer (10 mM Tris–HCl pH 7.5, 100 mM KCl, 20 mM MgCl_2_, 1× protease inhibitor (Roche)) and incubated with rotation at 10 rpm for 2 h at 4°C with 100 μl anti-FLAG M2 beads (Sigma-Aldrich, cat. no. A2220, equilibrated twice with 15 volumes TBS and wash buffer (dilution buffer supplemented with 0.1% digitonin)). Following binding, beads were washed three times with 15 volumes of wash buffer and two times with dilution buffer. Supernatants were removed and beads bound to co-immunoprecipitated samples were snap frozen in liquid nitrogen and stored at −80°C until further preparation for proteomic identification.

### Preparation of isolated mitochondrial samples for proteomic identification

Percoll-purified heart mitochondria (120 μg) were resuspended in 100 μl of 6 M guanidine chloride (Sigma-Aldrich, cat. no. G3272) and 100 mM Tris–HCl (pH 8.0). Samples were incubated at room temperature for 10 min, sonicated for 100 s in 10 s on/off cycles, spun down briefly, and incubated again for 10 min. Lysates were centrifuged for 10 min (max. speed) and protein concentration of the supernatant was determined by BCA assay (Pierce). Proteins were reduced with 5 mM 1,4-dithiothreiotol at 55°C for 30 min, cooled briefly on ice, and alkylated with 15 mM 2-chloroacetamide for 15 min in the dark. Fifty micrograms of protein quantified by BCA assay were diluted 1:10 in 50 mM Tris–HCl (pH 8.0) and digested overnight with 1 μg (1:50, protein:trypsin) of Pierce™ MS-grade trypsin (Thermo Fisher Scientific, cat. no. 90057) at 37°C and mild shaking. The digest was stopped by adding 1.2% formic acid and precipitates were removed by centrifugation at 3000g for 10 min. Peptides were desalted using Pierce™ Peptide Desalting Spin columns (Thermo Fisher Scientific, cat. no. 89851) following the manufacturer's instructions. Eluted peptides were dried at 30°C in a Speedvac concentrator (Eppendorf), and quantified in 0.5% formic acid using a NanoDrop (Thermo Fisher Scientific). Dried peptides were stored at −80°C until delivered to the mass spectrometry facility.

Pellets of percoll-purified liver mitochondria were thawed on ice and resuspended in lysis buffer (45 μl of 8M urea and 5 μl of 1% ProteaseMAX (Promega) in 100 mM Tris–HCl (pH 8.5) with protease and phosphatase inhibitors). Samples were sonicated in water bath for 10 min and probe sonicated using VibraCell probe (Sonics & Materials, Inc.) for 20 s with pulse 2/2, at 20% amplitude. Lysates were spun down at 12 000 rpm at 4°C for 10 min and protein concentration was determined by BCA assay (Pierce). An aliquot of 50 μg protein in 25 μl lysis buffer was supplemented with 20 μl of 100 mM Tris–HCl and 5 μl acetonitrile (ACN). Proteins were reduced by adding 1 μl of 0.5 M dithiothreitol (Sigma) and incubated at 25°C for 60 min while shaking at 400 rpm on a block heater. Alkylation was performed with addition of 3 μl of 0.5 M iodoacetamide (Sigma) at room temperature for 60 min and quenched with 2 μl of 0.5M dithiothreitol. Digestion was started with 4 μl of 0.5 μg/μl Lys-C in water incubating at 25°C for 4 h at 450 rpm shaking on a block heater before adding 150 μl of Tris–HCl, pH 8.5 and completing with 4 μl of 0.5 μg/μl sequencing grade modified trypsin (Promega) incubated for 16 h at 37°C. The digestion was stopped with 8 μl cc. formic acid, incubating the solutions at room temperature for 5 min. The samples were cleaned on a C18 Hypersep plate with 40 μl bed volume (Thermo Fisher Scientific), dried using a vacuum concentrator (Eppendorf).

### Preparation of co-immunoprecipitated samples for proteomic identification

Agarose beads from FLAG immunoprecipation were supplemented with 25 μl of 8M urea in 100 mM Tris–HCl (pH 8.0) and additional 120 μl of 100 mM Tris–HCl was supplemented, followed by adding 5 μl of 0.1 μg/μl trypsin (sequencing grade, Promega) and incubated at 27°C for 30 min with shake at 800 rpm. Beads were pelleted down, and the supernatant was collected (ca. 150 μl) in a new tube. The beads were stepwise washed with 25 μl of 1 mM DTT in 100 mM Tris–HCl, pH 8 twice and 50 μl of 100 mM Tris–HCl collecting the supernatants in the same tube (total ca. 350 μl). Digestion in the eluent was continued over night at room temperature adding 5 μl of 0.1 μg/μl trypsin with shaking at 400 rpm, while the agarose beads were supplemented with 50 μl of 100 mM Tris–HCl and further digested with 5 μl of 0.1 μg/μl trypsin overnight at RT with shaking at 400 rpm. The peptide solutions were alkylated with 0.5M iodoacetamide in 100 mM Tris–HCl, pH 8 incubating at RT for 30 min in dark. The digestion was stopped with 20 μl of cc. formic acid in the combined peptide solution and samples were cleaned on a C-18 HyperSep plate with 40 μl bed volume (Thermo Fisher Scientific) and dried in a vacuum concentrator (Eppendorf).

### Liquid chromatography–tandem mass spectrometry data acquisition

Peptides were reconstituted in 2% acetonitrile and 0.1% formic acid (17 μl) and 4.6 μl was injected on a 50 cm long EASY-Spray C18 column (Thermo Fisher Scientific) connected to an Ultimate 3000 nanoUPLC system (Thermo Fisher Scientific) using a 90 (IP samples) or 120 min (mitochondrial samples) long gradient: 4–26% of solvent B (98% acetonitrile, 0.1% FA) in 90/120 min, 26–95% in 5 min, and 95% of solvent B for 5 min at a flow rate of 300 nl/min. Mass spectra were acquired on a Q Exactive HF hybrid quadrupole orbitrap mass spectrometer (Thermo Fisher Scientific) ranging from *m/z* 375 to 1800 (IP samles) or 1500 (mitochondrial samples) at a resolution of *R* = 120 000 (at *m/z* 200) targeting 5 × 10^6^ ions for maximum injection time of 100 ms, followed by data-dependent higher-energy collisional dissociation (HCD) fragmentations of precursor ions with a charge state 2+ to 8+, using 45 s dynamic exclusion. The tandem mass spectra of the top 17 precursor ions were acquired with a resolution of *R* = 30 000, targeting 2 × 10^5^ ions for maximum injection time of 54 ms, setting quadrupole isolation width to 1.4 Th and normalized collision energy to 28%.

### Mass spectrometry data analysis

The raw LC–MS/MS data files were processed using MaxQuant (27) version 1.6.10.43 on an Ubuntu Linux 20.04.1 LTS server at the SNIC Science Cloud. The search database consisted of the canonical sequences of the UniProt mouse proteome UP000000589 as of 29 October 2019 (55176 proteins) and default settings were used with label free quantification, variable methionine oxidation and protein N-terminal acetylation, as well as fixed cysteine carbamidomethylation as modifications. Match-between-runs was enabled for the heart (mitoribosome) IP dataset. Protein group intensity values from MaxQuant passing the FDR <0.01 cutoff were further processed in R 4.1 markdown notebooks and first submitted to NormalyzerDE (28) for dataset overview and cyclic loess normalization. Protein groups were annotated as known mitochondrial using MitoCarta 3.0 (9) with gene name matching. Differential abundance analysis was done using LIMMA empirical bayes statistics (29) in NormalyzerDE. The mitoprep batch was included as covariate for all comparisons as batch effects were observed in PCA. The LC–MS/MS data have been deposited to the ProteomeXchange Consortium via the PRIDE (30) partner repository with the dataset identifier PXD046268. Volcano plots were generated using Rstudio version 1.4.1717.

### Comparisons between proteomic datasets

Venn diagrams were generated using FunRich Tool (version 3.1.4). Significantly reduced mitochondrial proteins in *Slirp*^−/−^ mitochondria from heart and liver were compared with complex I or complex IV subunits (Mouse MitoCarta 3.0) and with the significantly reduced proteins in *Lrpprc*^−/−^ heart mitochondria (31). Reduced proteins were considered significant with an adjusted *P*-value <0.1.

### Denaturing protein gel electrophoresis and western blots

Isolated mitochondria were resuspended in 2× NuPAGE™ LDS Sample Buffer in the presence of NuPAGE™ reducing agent (Invitrogen) and incubated at 70°C for 10 min. Mouse embryonic fibroblasts were trypsinized, pelleted and resuspended in RIPA buffer (50 mM Tris–HCl, 150 mM NaCl, 1% NP-40, 0.5% Sodium-deoxycholate, 0.1% SDS) in the presence of 1.25 U/μl Benzonase. Proteins were resolved by electrophoresis using 4–12% or 12% Bis–Tris Plus NuPAGE gels (Invitrogen) with MOPS or MES buffer. Precision Plus DNA ladder was used as a molecular weight marker. Gels were transferred using wet transfer or the iBlot 2 system (Invitrogen). Membranes were blocked in TBS-T/5% milk, incubated with the primary antibody and incubated with the secondary antibody in TBS-T/5% milk, with washes with TBS-T between incubations. Details of all antibodies used are provided in Supplementary Table S3. Membranes were treated with Clarity Western ECL substrate (BioRad) and western blots were developed in a darkroom or using a BioRad ChemiDoc. Band intensity was measured by densitometric analysis using the GelAnalyzer 2010a freeware.

### BN-PAGE, in-gel activity assays and second dimension gels

Isolated mitochondria (300 μg) were resuspended in 75 μl of solubilization buffer (1% *n*-dodecyl β-d-maltoside (2.5 g dodecyl maltoside/g protein), 20 mM Tris/HCl (pH7.4), 0.1 mM EDTA, 50 mM NaCl, 10% glycerol and 1 mM PMSF). Samples were incubated on ice for 20–30 min and centrifuged for 10 min at 14 000 rpm at 4°C. Supernatants were recovered into fresh tubes, and 7.5 μl of loading dye (5% w/v Coomassie Brilliant Blue G-250, 100 mM Bis–Tris pH 7.0 and 500 mM 6-aminocaproic acid) were added to each sample. Samples (50–125 μg) were resolved in precast Bis-tris blue native gels (Invitrogen NativePAGE 4–16%). Gels were stained with Coomassie or in-gel activity was assayed by incubating gels in specific substrates for complex I (2 mM Tris–HCl, pH 7.4, 0.1 mg/ml NADH, 2 mg/ml iodonitrozolium), complex IV (50 mM phosphate buffer, pH 7.4, 0.5 mg/ml 3,3′-diamidobenzidine tetrahydrochloride, 1 mg/ml cytochrome *c*, 220 mM sucrose and 0.02 mg/ml catalase) and ATPase activity of complex V (first, the gel was washed with 50 mM glycine, 5 mM MgCl_2_, 0.1% Triton X-100, 0.5 mg/ml lead acetate, pH 8.0 (with NaOH), then the buffer was replaced for fresh one supplemented with 4 mM ATP). For second dimension gels, individual lanes were excised after native electrophoresis, incubated in MOPS buffer for 10 min and stacked on denaturing 12% Bis–Tris Plus NuPAGE gels (Invitrogen). For western blotting, second dimension gels were incubated in Towbin buffer for 10 minutes and transferred to PVDF membranes using the iBlot2 system (Invitrogen). After transfer, gels were stained with Coomassie. Gels with [^35^S]-labelled proteins were stained with Coomassie and dried as described below.

### [^35^S] methionine labelling of mitochondrial translation products

For *in organello* labelling, 500 μg of isolated mitochondria (1 mg if pulse + chase, all amounts were doubled) were washed twice (all centrifugation steps were at 9000g for 10 min at 4°C) with 500 μl of translation buffer containing translation mix (100 mM Mannitol, 10 mM Na-succinate dibasic hexahydrate, 80 mM KCl, 5 mM MgCl_2_ hexahydrate, 1 mM KH_2_PO_4_, 25 mM HEPES, pH7.4), 60 μg/ml of 19 of the 20 canonic l-aminoacids (all except methionine), 5 mM ATP, 200 μM GTP, 6 mM phosphocreatine, 60 μg/ml creatine phosphate, 200 μg/ml emetine and 100 μg/ml cycloheximide. The pellet was resuspended in 750 μl of translation buffer, 150 μCi of L-[^35^S]-methionine (PerkinElmer, cat. no. NEG709A) were added (if pulse + chase, separate samples into two tubes), and samples were incubated for 1 h at 37°C in rotation followed by two washes with 500 μl of translation buffer. If needed, chase tubes were resuspended in 750 μl of translation buffer supplemented with 60 μg/μl of cold methionine and incubated for the indicated times at 37°C in rotation followed by two washes with 500 μl of translation buffer. Pellets were resuspended in 40–50 μl of 2× LDS-Sample buffer (with reducing agent) and incubated at room temperature for 30 min or in solubilization buffer for BN-PAGE.

For [^35^S] labelling of mitochondrial translation in mouse embryonic fibroblasts, 80–90% confluent cells in 6-well plates were washed with PBS and incubated twice with 2 ml of Cystine- and Methionine-free DMEM media (Gibco Cat. No. 21013024) for 5 min at 37°C. Then, each well was incubated with 1 ml of labeling media (Cystine- and Methionine-free DMEM media supplemented with 10% dialyzed fetal bovine serum (FBS), 1 mM sodium pyruvate, 1× GlutaMAX-1 (Gibco) and 125 μg/ml emetine) for 20 min at 37°C. Media was then replaced with 1 ml of the same media supplemented with 35.2 μCi/ml of [^35^S] methionine and cysteine mix (PerkinElmer, NEG772002) and cells were incubated for 25 min at 37°C. Cells were washed three times with ice-cold PBS, harvested by trypsinization and centrifugation at 1500g for 5 min at 4°C. Pellets were solubilized in 50 μl of RIPA buffer (supplemented with 1.25 U/μl Benzonase and protease inhibitors), incubated on ice for 30 min and cell debris was pelleted by centrifugation. Protein concentration of the supernatants was determined using the Qubit system and samples for SDS-PAGE were prepared in NuPAGE™ LDS Sample Buffer.

After SDS-PAGE running, gels were stained with Coomassie, incubated in a solution of 30% methanol and 3% glycerol, and dried on Whatmann paper under vacuum at 60°C for 1.5 h. Radioactive signals were captured in a PhosphorImager screen, and screens were scanned using a Typhoon FLA 7000 and the ImageQuant TL 8.1 software (GE Healthcare).

### RNA isolation and preparation of cDNA

Total RNA was isolated from snap-frozen tissue biopsies using the TriZol/chloroform extraction and isopropanol precipitation method. Samples were treated with DNase-I (Turbo DNA-free kit, Ambion) according to the manufacturer's instructions and RNA concentrations were measured using a Nanodrop. Total RNA from mouse embryonic fibroblasts was obtained using the RNeasy Mini kit and the RNase-free DNase set (Qiagen) according to the manufacturer's instructions. DNase-treated RNA (1–2 μg) was used for a reverse transcription reaction using the High-Capacity cDNA Reverse Transcription Kit (Applied Biosystems) according to the manufacturer's instructions.

### Mitochondrial transcript measurements by RT-qPCR and northern blots

RT-qPCRs were run in a QuantStudio 6 Flex Real-Time PCR System using TaqMan™ Universal Master Mix II, with UNG and Taqman probes. *Actin* was used as a loading control. Each reaction was prepared in triplicate and a Delta Ct analysis was performed. Details of qPCR probes are provided in Supplementary Table S3.

For northern blotting, DNase-treated RNA (2 μg in 12 μl) was separated in 1.2% agarose, 1× NorthernMax MOPS, 18% formaldehyde gels. Separated RNAs were then transferred by capillarity to Hybond-N+ membranes (0.45 μm, Amersham) and the membrane was UV-crosslinked with 4000 × 100 μJ/cm^2^. Individual transcripts were detected by hybridizing the membranes with [^32^P] dCTP-labeled dsDNA probes, following the Prime-It II Random Primer Labeling Kit (Agilent). Details of all templates used to prepare northern blot probes are provided in Supplementary Table S3. Radioactive signals were captured in a PhosphorImager screen, and screens were scanned using a Typhoon FLA 7000 and the ImageQuant TL 8.1 software (GE Healthcare).

### Poly(A) tail length measurement of mt-mRNAs by 3′ RACE

The assay was based and altered from previous protocols (14,32,33). DNase-treated RNA (5 μg) was ligated to 100pmol of 5′ phosphorylated oligonucleotide linker by T4 RNA ligase 1 (New England Bioloabs) at 25°C for 2h, according to the manufacturer's instructions. Ligated RNA was TriZol-extracted, isopropanol-precipitated and reverse transcribed using the High-capacity cDNA reverse transcription kit (Applied Biosystems) using a primer complementary to the linker sequence (anti-linker). The 3′ end of each mt-mRNA was amplified by PCR using the anti-linker and gene-specific primers, and the PCR products were resolved in 2.5% agarose gels. To confirm the amplification of the desired products, bands of interest were gel-purified with the QIAquick Gel Extraxtion kit (QIAGEN), cloned into pCR4-TOPO vectors and transformed into One Shot TOP10 competent cells (Thermo Scientific). Plasmid DNA was extracted and sequenced using the M13 primer. All oligonucleotide sequences are listed in Supplementary Table S3.

### DNA isolation and mtDNA level measurements

Genomic RNA-free DNA from snap frozen mouse tissues was isolated using the DNeasy Blood and Tissue Kit (Qiagen) according to manufacturer's instructions including treatment with RNase A. Relative mtDNA levels were determined by qPCR using 5 ng of DNA in a QuantStudio 6 Flex Real-Time PCR System. *18S* was used as loading control. Each reaction was prepared in triplicate and a Delta Ct analysis was performed. Details of qPCR probes are provided in Supplementary Table S3.

### Sucrose gradient profiles

Heart and liver mitochondria (1.2 mg) were solubilized in 120 μl of lysis buffer (260 mM sucrose, 10 mM Tris–HCl pH 7.5, 100 mM KCl, 20 mM MgCl_2_, 1× protease inhibitor (Roche) and 1% *n*-dodecyl β-d-maltoside) for 20 min on ice. Cleared lysates were loaded on 10–30% sucrose gradients (prepared with ice-cold solutions) and centrifuged for 15 h at 71 000g at 4°C. Gradient fractions were collected as 750 μl aliquots. RNA was extracted from one third (250 μl) of each fraction using TRIzol LS Reagent (Invitrogen) according to the manufacturer's instructions and treated with DNase I. Then, cDNA for each fraction was synthesized from 5 μl of RNA. Transcript abundance in each fraction was assessed by qRT-PCR using Taqman probes (Supplementary Table S3) and standard curve analysis setup in a QuantStudio 6 system. The rest of the DNA-free RNA was prepared for northern blotting. Protein was precipitated from another third of each fraction using trichloroacetic acid, resolved by SDS-PAGE and protein levels from all fractions were detected by western blotting.

### Mouse embryo cryo-sectioning and COX/SDH histochemistry

Embryos from mice at day E13.5 were dissected, embedded in optimal cutting temperature compound (OCT), and cryo-sectioned at mid-sagittal regions (12 μm thick on Poly-L-Lysine slides). COX/SDH staining was done by incubating slides for 50 min at 37°C in freshly prepared COX solution (100 μM cytochrome *c*, 4 mM 3,3 diaminobenzidine tetrahydrochloride, catalase (20 μg/ml), and 100 mM phosphate buffer (pH 7.0)), slides were washed three times in PBS. Next, slides were incubated for 40 min at 37°C in freshly prepared SDH medium (130 mM sodium succinate, 200 μM phenazinemethosulphate, 1 mM sodium azide, 1.5 mM nitroblue tetrazolium and 100 mM phosphate buffer (pH 7.0)). Slides were washed three times in PBS, dehydrated with increasing ethanol concentrations (70%, 95%, 99%), and mounted for bright-field microscopy. Total areas of sections were calculated using the QuPath software.

### Quantification of the m.C5024T mutation heteroplasmy levels

The m.C5024T mutation heteroplasmy was measured using a QIAGEN PyroMark Q24 pyrosequencer as previously described (25). In short, sample DNA was obtained by alkaline extraction and a 178-bp fragment spanning the m.5024 mutation site was amplified by PCR using a biotinylated forward primer (forward: 5′-Biotin-TTCCACCCTAGCTATCATAAGC, reverse: 5′-GTAGGTTTAATTCCTGCCAATCT). Sequencing was carried out with PyroMark Q24 Advanced Reagents, Streptavidin Sepharose TM high-performance beads (GE Healthcare), and the sequencing primer (TGTAGGATGAAGTCTTACA) according to the manufacturer's instructions.

### Identification of differentially expressed genes in m.C5024T embryos

Analysis of differentially expressed genes was performed as previously described (34). In brief, raw counts were processed using Seurat (v.4.0.5) and SCTransform (sctransform R package, v.0.3.2) with regressing out batch effect, followed by Harmony (v.0.1.0). Cell-type-and-genotype-specific differentially expressed genes were calculated with Seurat function ‘FindMarkers’, using a Wilcoxon rank-sum test, with log2(fold change) threshold of ±0.25, significance threshold of 0.05 and use of the Benjamini-Hochberg procedure to obtain multiple-testing corrected *P* values. Data were plotted using GraphPad Prism version 9.5.1 for MacOS (GraphPad Software, Boston, Massachusetts USA, www.graphpad.com).

### Preparation of mouse embryonic fibroblasts

Each mouse embryo (E13.5) was washed in sterile PBS, dissected from red organs and head, and thinly diced. Fibroblasts were dissociated in 1 ml of trypLE Express reagent (Gibco) and incubated for 30 min at 37°C. Trypsin was inactivated with DMEM media (Gibco cat. no. 10569010) supplemented with 10% FBS and 1% penicillin-streptomycin (Gibco) and fibroblasts were transferred to a culture flask and incubated at 37°C.

### Cell proliferation curves

Mouse embryonic fibroblasts were seeded in 96-well plates (2000 cells/well, one plate/timepoint) in the presence of media containing glucose (DMEM cat. no. 10569010, 10% FBS, 1% penicillin-streptomycin) or galactose (DMEM, no glucose Gibco cat. no. 11966025, 15 mM galactose, 1 mM pyruvate, 10% FBS, 1% penicillin-streptomycin, 1% non-essential amino acids, 50 μg/ml uridine). Cell proliferation was determined by colorimetry using the cell counting kit 8 (WST-8, ab228554).

## Results

### The absence of SLIRP leads to decreased complex I protein levels

**Figure 1.**
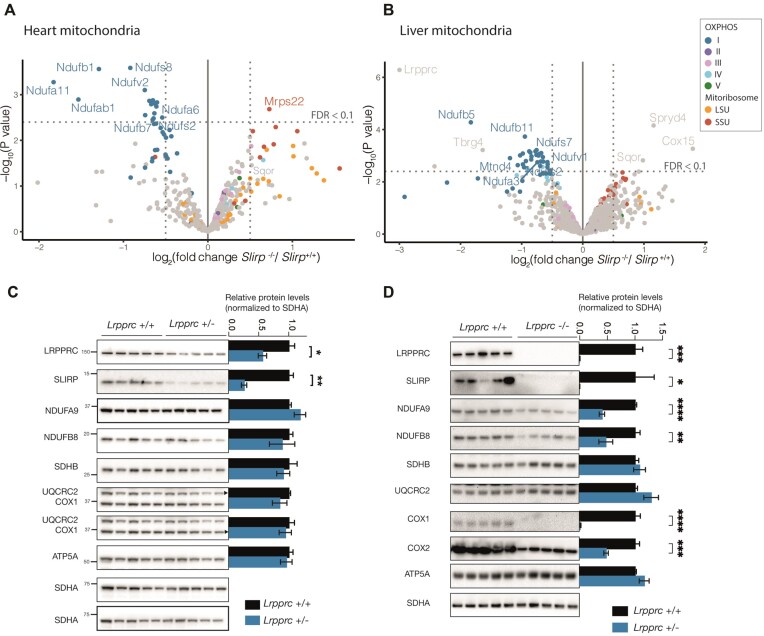
A drastic reduction in LRPPRC/SLIRP levels leads to a decrease in complex I protein levels. (A, B) Volcano plots of comparisons of heart (**A**) and liver (**B**) mitoproteomes (*n*= 5). Percoll-purified mitochondria of 12-week-old *Slirp* knockout (*Slirp*^−/−^) and wildtype (*Slirp*^+/+^) mice were submitted to proteomic identification. The dashed line marks the false discovery rate cut-off (FDR < 0.1), corresponding gene names of proteins above the cut-off value are labeled. OXPHOS and mitoribosome subunits are colour-coded. LRPPRC is not plotted in heart because it was only detected in one replicate of *Slirp*^−/−^ samples and no *P*-value was calculated. (**C**) Western blots of steady-state levels of LRPPRC, SLIRP, and OXPHOS subunits in heart mitochondria (10 ug) from 12-week-old control (*Lrpprc*^+/+^) and tissue-specific *Lrpprc* heterozygous knockout (*Lrpprc*^+/−^) mice. SDHA was used as a loading control. Densitometric quantification is shown for each blot, error bars are SEM, *n* = 5 biological replicates. *P value < 0.05, **P value < 0.01. (**D**) Western blots of steady-state levels of LRPPRC, SLIRP, and OXPHOS subunits in heart mitochondria (10 ug) from 12-week-old control (*Lrpprc*^+/+^) and tissue-specific *Lrpprc* knockout (*Lrpprc*^−/−^) mice. SDHA was used as a loading control. Densitometric quantification is shown for each blot, error bars are SEM, *n* = 5 biological replicates. *P value < 0.05, **P value < 0.01, ***P value < 0.001, ****P value < 0.0001.

We characterized the mitochondrial proteome of *Slirp*^−/−^ mice by subjecting Percoll-purified mitochondria to label-free quantitative proteomics and found decreased levels of complex I subunits in both heart and liver whereas the protein levels of other OXPHOS complexes are normal (Figure 1A, B, Ext. Figure 1A, B, Supplementary Table S1). Most mitoribosome SSU proteins tend to increase whereas LSU proteins show moderate changes in both directions. We have previously shown that mt-rRNAs are upregulated in *Slirp*^−/−^ mice (14). Our proteomic data shows that this upregulation in mt-rRNAs does not directly result in a proportional increase in mitoribosome abundance. As protein levels of LRPPRC drop to ∼25% of normal levels in the absence of SLIRP (14), we investigated whether a less drastic decrease of LRPPRC and SLIRP also affects levels of complex I subunits. In tissue-specific *Lrpprc*^+/−^ heart mitochondria, the levels of LRPPRC are ∼50% and levels of SLIRP are ∼25% of normal levels but western blots to detect complex I subunits showed no changes (Figure 1C). This suggests that only a drastic reduction in LRPPRC/SLIRP levels will lead to a decrease in levels of complex I subunits. Heart-specific *Lrpprc*^−/−^ mitochondria present a comparable decrease in complex I subunits although this model causes further proteomic changes including a marked decrease in complex IV subunits that result in a more marked phenotype (Figure 1D, Ext. Figure 1C, D) (31). We have previously shown that *Slirp*^−/−^ mice have no clear decrease in complex I enzyme activity and are essentially healthy despite a small increase of lactic acid in blood (14).

Next, we crossed *Slirp*^−/−^ mice with the MitoRiboTag (mL62-Flag) mouse model (26) to detect changes in the protein-protein interactions of immunoprecipitated mitoribosomes when SLIRP is absent and LRPPRC depleted (Ext. Figure 1E, F, Supplementary Table S1). Using quantitative proteomics of immunoprecipitated mitoribosomes, we identified almost the complete set of mitoribosomal proteins (78 of 82 proteins, Supplementary Table S2) in both heart and liver samples. We also found a decrease in levels of complex I subunits (Figure 1C, D) consistent with the results from proteomics of whole mitochondria (Figure 1A, B). We did not observe significant (FDR < 0.1) enrichment differences that would indicate changes to the mitoribosome interactomes when SLIRP is absent, indicating that a decrease in mt-mRNAs does not result in marked changes to the interactome of mitoribosomes *in vivo*. Factors involved in mitoribosome biogenesis and translation, namely MTIF3, GFM2, and MALSU1, were increased at lower stringency (FDR < 0.22) in liver. Consistent with this result, we have previously reported a clear defect in mitochondrial translation in liver when SLIRP is absent (14). Our results suggest that liver mitochondria elicit a potential response mechanism by increasing mitoribosome biogenesis.

We found an increase in SQOR, a CoQ-linked enzyme of the sulphide oxidation pathway, in our datasets. SQOR is also reported to accumulate in brain of *Ndufs4*^−/−^ mice (35). Further studies are needed to gain insights into the potential connection between complex I biogenesis, iron-sulphur clusters, SQOR regulation, and coenzyme Q metabolism.

Taken together, our proteomic analyses of *Slirp*^−/−^ mitochondria from heart and liver revealed a clear decrease of complex I protein subunits. However, the decrease in mt-mRNAs caused by the lack of SLIRP does not elicit marked changes to the interactomes of mitoribosomes *in vivo*.

### *In vivo* disruption of the interaction between LRPPRC and SLIRP results in complete degradation of SLIRP

**Figure 2.**
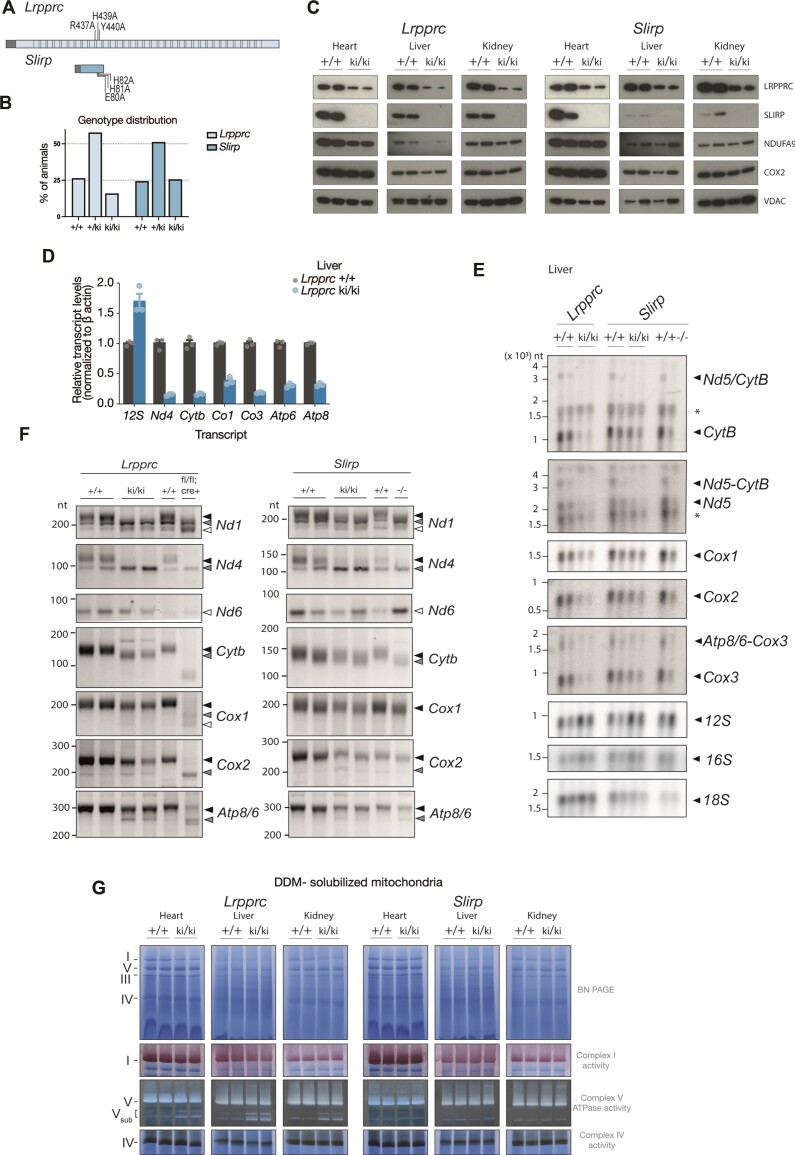
Disruption of the interaction of LRPPRC and SLIRP *in vivo*. (**A**) Graphic representation of the residues of LRPPRC and SLIRP that were substituted in the knock-in lines (*Lrpprc*^ki/ki^ and *Slirp*^ki/ki^). Mitochondria targeting sequences are depicted as grey boxes. PPR domains of LRPPRC are depicted as empty boxes. (**B**) Bar graph of the of percentages observed for each genotype in the offspring from the *Lrpprc* and *Slirp* knock-in lines, dotted lines indicate the expected percentages. A chi-square test rejects that the observed genotypes in the *Lrpprc*^ki/ki^ knock-in line follow the expected mendelian distribution, significance level (α) 0.02. (**C**) Western blot of steady-state protein levels of LRPPRC, SLIRP, NDUFA9 and COX2 in heart, liver, and kidney mitochondria (5 μg) from 8-week-old control (+/+) and *Lrpprc*^ki/ki^ or *Slirp*^ki/ki^ mutants. Porin (VDAC) was used as a loading control. (**D**) RT-qPCR analyses of mitochondrial transcript levels in liver mitochondria from 8-week-old control (+/+) and *Lrpprc*^ki/ki^ mice, *n* = 3 biological replicates. (**E**) Northern blot analyses of mt-mRNA and mt-rRNA transcripts in 8-week-old *Lrpprc*^ki/ki^, and *Slirp*^ki/ki^ and *Slirp*^−/−^ mouse livers. 18S rRNA was used as loading control. (**F**) Polyadenylation profiles of mt-mRNAs in *Lrpprc*^ki/ki^ and *Slirp*^ki/ki^ lines by 3′RACE assays. RT-PCR products derived from polyadenylated, oligoadenylated or non-adenylated RNAs are indicated by black, gray, and white triangles, respectively. *Lrpprc*^−/−^ heart and *Slirp*^−/−^ liver samples were included for comparison. (**G**) In-gel activity stainings of OXPHOS complexes I, IV and V. Heart and liver mitochondria from *Lrpprc*^ki/ki^ and *Slirp*^ki/ki^ mice were solubilized with dodecyl-maltoside and resolved by blue-native gel electrophoresis (BN-PAGE) followed by in-gel activity.

We proceeded to investigate whether heterodimer formation is important for the *in vivo* functions of LRPPRC and SLIRP in promoting mt-mRNA stability, transcript maturation, and mitochondrial translation. To this end, we generated two CRISPR-Cas9 knock-in mouse lines, each carrying mutations at the binding interphases of either LRPPRC or SLIRP. We have previously identified the key residues necessary for heterodimer formation between LRPPRC and SLIRP (20), and we now used this information to create a *Lrpprc*^ki/ki^ line with three residue substitutions on LRPPRC (R437A, H439A, and Y440A) and a *Slirp*^ki/ki^ line with three substitutions on SLIRP (E80A, H81A, H82A, Figure 2A). Crosses of heterozygous mice produced pups of all expected genotypes (+/+, +/ki and ki/ki), but *Lrpprc*^ki/ki^ mice were obtained at less than expected mendelian ratios (∼16% of *Lrpprc*^ki/ki^ mice instead of expected ∼25%) (Figure 2B). The mean litter size of the *Lrpprc* line was lower compared with the *Slirp* line but we observed no increased frequency of pups dying after birth, suggesting prenatal lethality of a fraction of the homozygous *Lrpprc*^ki/ki^ mice (Ext. Figure 2A). Born homozygous knock-in mice from both lines were apparently healthy and did not show any defects in weight gain or heart to body weight ratio (data not shown).

SLIRP was undetectable in isolated mitochondria from heart, liver, and kidney from both homozygous knock-in lines (Figure 2C). We synthesized recombinant wild-type (SLIRP^WT^) and mutant (SLIRP^KI^) protein and validated that the antibody recognizes both versions of the protein (Ext. Figure 2B). The levels of *Slirp* and *Lrpprc* transcripts were unchanged in both knock-in lines (Ext. Figure 2F), arguing that the synthesis of SLIRP^KI^ and LRPPRC^KI^ is unaffected. Both LRPPRC and SLIRP have amino-terminal mitochondrial targeting sequences (MTS) that are cleaved after import to the mitochondrial matrix. It is important to recognize that the amino acid substitutions in LRPPRC^KI^ or SLIRP^KI^ do not involve the MTS (Figure 2A) and there is therefore no reason to believe that the corresponding proteins cannot be imported. Furthermore, we performed western blots in whole-tissue extracts from hearts (Ext. Figure 2C) and observed that, similar to the western blots of isolated mitochondria, LRPPRC levels were reduced and SLIRP not detected in both knock-in mouse lines. These results thus argue against accumulation of the mutated proteins in the cytosol. Finally, we would like to point out that in the *Lrpprc*^ki/ki^ line, the SLIRP protein has a wildtype sequence (SLIRP^WT^) and is nevertheless absent in mitochondria. The most like explanation for this finding is that the SLIRP^WT^ protein is normally synthesized in the cytosol and imported into mitochondria where it is degraded. The degradation is likely due to its lack of stabilization as it cannot bind the mutant LRPPRC (LRPPRC^KI^) in the mitochondrial matrix. Taken together, we conclude that SLIRP is tightly controlled and completely degraded in the mitochondrial matrix upon losing its interaction with LRPPRC. These results confirm that the residues that mediate the interaction of LRPPRC and SLIRP in a recombinant system are essential for their interaction also *in vivo*.

We observed that the LRPPRC levels were decreased in both knock-in lines and were further affected if LRPPRC carried the knock-in substitutions (Figure 2C, and Ext. Figure 2D), likely because the substitutions also affect the stability of LRPPRC. Indeed, mt-mRNAs were down and mt-rRNAs were up in tissues from both homozygous knock-in lines with the most marked decreases in *Lrpprc*^ki/ki^ samples (Figure 2D, E and Ext. Figure 2F). The molecular phenotype of *Slirp*^ki/ki^ mice was generally milder than *Lrpprc*^ki/ki^ and *Slirp*^−/−^ knockout mice, e.g. regarding the extent of decrease in complex I subunits (Ext. Figure 2E) and mt-mRNA levels (Figure 2E).

To explore the polyadenylation status of mt-mRNAs, we performed 3′RACE assays of isolated liver RNA from both knock-in lines. Poly(A) length was decreased in *Lrpprc*^ki/ki^ samples although to a lesser extent than the decrease observed in *Lrpprc*^−/−^ heart samples (Figure 2F). The polyadenylation of mt-mRNAs from the *Slirp*^ki/ki^ line was mildly decreased (*Nd1* and *Nd4*) or unchanged (*Cox1* and *Atp8/6*).

Next, we resolved mitochondrial OXPHOS complexes using native electrophoresis (BN-PAGE) to assess the in-gel activities of complexes I, IV and V. *Lrpprc*^ki/ki^ mitochondria show a decrease in levels of assembled complex I (Figure 2G), whereas the BN-PAGE in-gel complex I activity assay show no clear difference likely because it is not sensitive enough. Fully assembled complexes IV and V and their in-gel activities were unaffected in *Lrpprc*^ki/ki^ and *Slirp*^ki/ki^ samples from heart, liver, and kidney (Figure 2F). Additionally, the knock-in substitutions in LRPPRC induced an increase in levels of subassembly intermediates of ATP synthase with ATPase activity. The ATPα subunit (of the F_1_ portion) was present in both the assembled and sub assembled complexes, and ATP8 (of the F_O_ portion) was observed only in the fully assembled ATP synthase complex (Ext. Figure 2G). ATP synthase intermediates containing F_1_ subunits are observed when mtDNA gene expression is compromised, for example in heart-specific *Lrpprc*^−/−^ mice (31,36) and have been previously characterized (37).

To summarize, if LRPPRC and SLIRP do not interact *in vivo*, LRPPRC is partially degraded and SLIRP is undetectable. When SLIRP carries the substitutions that disrupt binding to LRPPRC, the molecular phenotype of the mice is rather mild. When LRPPRC carries substitutions disrupting binding to SLIRP, the knock-in mice exhibit additional defects compared with *Slirp*^−/−^ and *Slirp*^ki/ki^ mice, i.e. the genotypes of the offspring deviate from mendelian ratios, mt-mRNAs are further decreased, poly(A) tail lengths of mt-mRNAs decrease, and levels of subassembly intermediates of complex V are elevated.

### Knock-in mutations in LRPPRC result in a defect in the translation of ATP8

**Figure 3.**
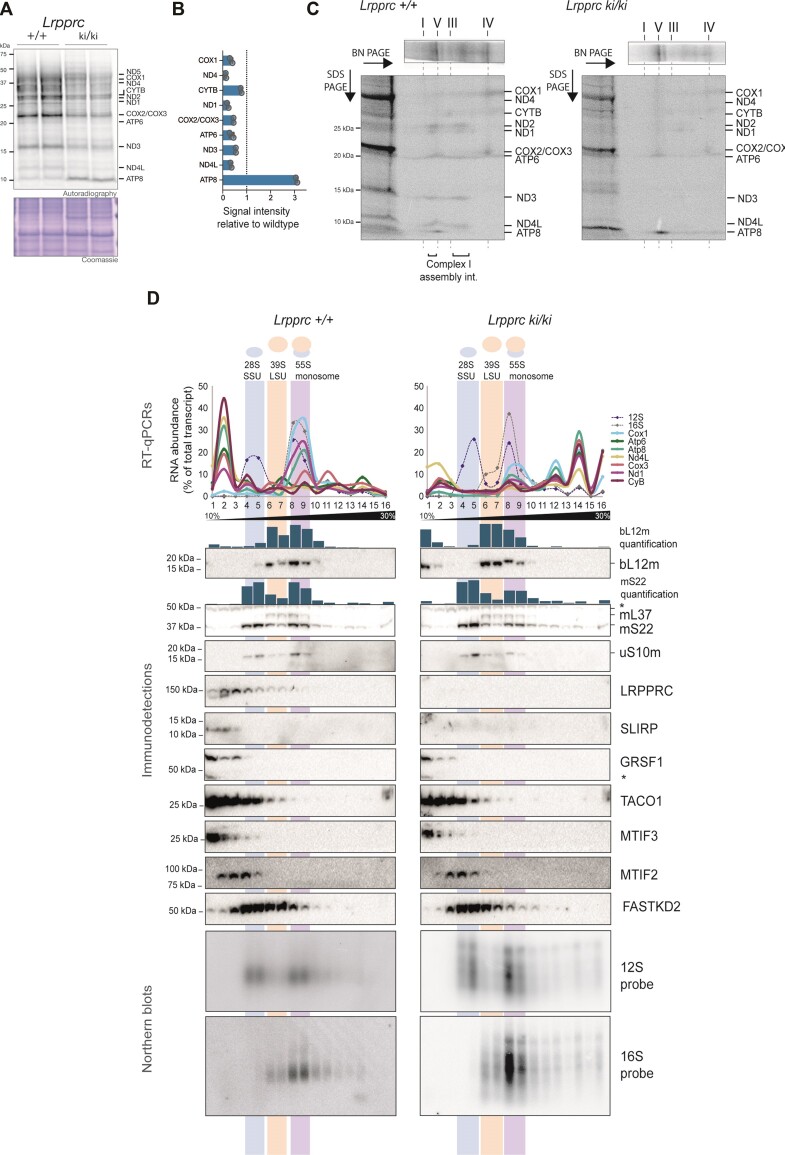
Translation profile of *Lrpprc*^ki/ki^ liver mitochondria. (**A**) Mitochondrial translation analysed by *in organello* [^35^S]-methionine labelling, LDS-PAGE, and digital autoradiography in liver mitochondria isolated from control (+/+) and *Lrpprc*^ki/ki^ 36-week-old mice, *n* = 6 replicates for each group (from 14–36 weeks old). Coomassie staining was used as a loading control. (**B**) Quantification of signal intensity relative to wildtype control of mitochondrial translation products shown in (A). (**C**) Autoradiography of second dimension electrophoresis of mitochondrial translation products. Newly synthesized proteins were labelled for 1h in the presence of [^35^S]-methionine followed by a 40-min chase in liver mitochondria isolated from control (+/+) and *Lrpprc*^ki/ki^ 14-week-old mice. Mitochondria were solubilized with dodecyl-maltoside and resolved by BN-PAGE (first dimension). Each lane was excised and further resolved by LDS-PAGE (second dimension). The first well of the second-dimension gel was loaded with mitochondria (75 μg) in LDS sample buffer. The positions of the fully assembled OXPHOS complexes I, III, IV and V are indicated in the first-dimension gel and mtDNA-encoded subunits are indicated in the second-dimension gel. The proposed position of a Complex I assembly intermediate is labelled. (**D**) Sedimentation profiles in sucrose gradients (10–30%) for individual mt-mRNAs detected by RT-qPCR (upper panel) in liver mitochondria isolated from control (+/+) and *Lrpprc*^ki/ki^ mice. The plotted percentage for each transcript is normalized to the total abundance for that transcript across all 16 fractions. Data of a representative experiment (from three independent experiments). Immunodetections and quantifications of the indicated proteins are shown in the middle panels, stars (*) indicate unspecific bands. Northern blots of all fractions hybridized with [^32^P]-dCTP-labelled probes recognizing 12S and 16S mt-rRNAs (bottom panels).

To evaluate if protein synthesis is compromised in the knock-in lines, we tracked mitochondrial translation by radiolabeling newly synthesized proteins with ^35^S-methionine in isolated mitochondria. Similar to *Slirp*^−/−^ tissues, mitochondrial translation in *Slirp*^ki/ki^ knock-in mice was markedly decreased in liver whereas changes in heart were not observed (Ext. Figure 3A, B). Translation in *Lrpprc*^ki/ki^ heart mitochondria showed mild changes compared to the control (Ext. Figure 3C, D), thus not recapitulating the chaotic translation observed when LRPPRC is completely absent (11). Interestingly, translation in *Lrpprc*^ki/ki^ liver mitochondria was decreased in general except for an increase in a ∼10 kDa band corresponding to the migration of ATP8 (Figure 3A, B). To further investigate if the ∼10kDa band corresponds to ATP8, we performed a second-dimension electrophoresis of mitochondrial translation products labelled with ^35^S-methionine. With this approach the assembled OXPHOS complexes are first resolved in native conditions in the first dimension followed by separation of the individual subunits in denaturing conditions in the second dimension. Radiolabeled ATP synthase complexes were fully assembled within the timeframe of the experiment and, in the second dimension, we identified that ATP8 corresponded to the overproduced ∼10 kDa protein in *Lrpprc*^ki/ki^ liver mitochondria (Figure 3C, Ext. Figure 3E, F). After a 2-h chase of the ^35^S-methionine pulse, the turnover of ATP8 was proportional to the rest of the labeled proteins (Ext. Figure 3G, H), showing that ATP8 is over-synthesized rather than stabilized.

The steady-state levels of the *Atp8/Atp6* bicistronic mt-mRNA were decreased like other protein-encoding mt-mRNAs (Figure 2D), but the steady-state levels of ATP8 protein were unchanged on western blots (Ext. Figure 3I). The LRPPRC protein is important for coordination of translation (11) and is involved in delivering mRNAs to the mitoribosome (24). When this translational coordination is impaired by the knock-in mutation of LRPPRC, the first open reading frame in the bicistronic *Atp8/Atp6* mRNA is preferentially translated and initiation of translation of the second open reading frame encoding ATP6 is reduced (Figure 3A, B).

We proceeded to investigate how mt-mRNAs interacted with the mitoribosome in *Lrpprc*^ki/ki^ mice. We layered solubilized mitochondria on sucrose gradients, ultracentrifuged and fractionated the gradients, and isolated RNA and protein from each fraction for gradient profiling by RT-qPCR and western blotting (Figure 3D). Small mitoribosomal subunits (28S SSU) were detected in fractions 4 and 5, as assessed by the presence of 12S rRNA, uS10m and mS22, proteins. Large mitoribosomal subunits (39S LSU) were detected in fractions 6 and 7, as assessed by the presence of 16S rRNA, bL12m and mL37 proteins. Mitochondrial 55S monosomes were detected in fractions 8 and 9. In *Lrpprc*^+/+^ liver mitochondria, mt-mRNAs were mostly detected in the monosome fractions and in fraction two, which corresponds to the pool of untranslated mt-mRNAs. In line with this, LRPPRC and SLIRP also sedimented at the top fractions of wildtype samples consistent with previous reports (11). In contrast, untranslated and monosome-associated mt-mRNAs were markedly decreased in *Lrpprc*^ki/ki^ samples from liver. Instead, a large proportion of the mt-mRNAs sedimented after the monosome fractions and was detected as separate peaks towards the bottom part of the gradient (fractions 12, 14 and 16). The profile of *Lrpprc*^ki/ki^ fractions from heart were more like the wildtype, the pool of untranslated mt-mRNAs was present but decreased, and a portion of mt-mRNAs sedimented at the bottom of the gradient (Ext. Figure 3J). To test if this abnormal sedimentation pattern is also observed in another model where mt-mRNAs are decreased, we performed sucrose gradients in *Slirp^−/−^* liver mitochondria (Ext. Figure 3K). Here, mt-mRNAs were also detected as separate peaks towards the bottom of the gradient.

Processing of mt-mRNAs in *Lrpprc*^ki/ki^ liver samples was unaffected and levels of unprocessed transcript intermediates were decreased proportionally to the mature forms (Figure 2E), showing that the shift in sedimentation in the gradient is not due to a higher molecular weight of the transcripts. To identify if liver mt-mRNAs had a shift in sedimentation in the gradient due to interactions with RNA binding proteins or translation factors, we immunodetected GRSF1, TACO1, MTIF3, MTIF2 and FASTKD2 (Figure 3D). Interactions with these proteins did not account for the shift in sedimentation of mt-mRNAs towards the bottom fractions of the gradient. Next, we confirmed the presence of 12S and 16S RNA peaks in fractions 12, 14 and 16 by northern blotting with radiolabeled probes (Figure 3D). Although we observe increased formation of a pattern consistent with the presence of polysomes when liver mitochondria from *Lrpprc*^ki/ki^ and *Slirp*^−/−^ mice are analyzed, only a minority of all mitoribosomes are involved. The absolute abundance of rRNAs is at least one order of magnitude higher than the abundance of mRNAs (6,10) and therefore only a minority of the ribosomes in wild-type and mutant samples will bind an mRNA. The northern and western blots show that a minority of 12S and 16S rRNA, and only a minor proportion of the mS22 protein are present in a pattern consistent with polysome formation (Figure 3D). However, there is a clear shift in mRNA distribution in the *Lrpprc*^ki/ki^ and *Slirp*^−/−^ liver mitochondria meaning that most mRNAs will be present in the polysome fraction, and a smaller proportion bound to monosomes (Figure 3D, Ext. Figure 3K). It is unclear why decreased levels of LRPPRC/SLIRP will result in polysome formation. We propose that without LRPPRC/SLIRP function the mRNAs will not only be present at lower levels but their interaction with the mitoribosome will occur in a non-coordinated fashion.

Taken together, our characterization of mitochondrial translation in *Lrpprc*^ki/ki^ tissues uncovered a distinctive defect where overall protein synthesis is decreased in liver, except for the increased synthesis of ATP8. Interestingly, we also detected peaks of mitoribosomes sedimenting at fractions after the monosome in a pattern characteristic of polysome formation. Thus, low LRPPRC/SLIRP levels reduce the steady-state levels of mt-mRNAs and causes poor coordination of mitochondrial translation leading to the binding of multiple mitoribosomes on single transcripts.

### SLIRP is essential in a mouse model harboring a pathogenic mtDNA mutation

**Figure 4.**
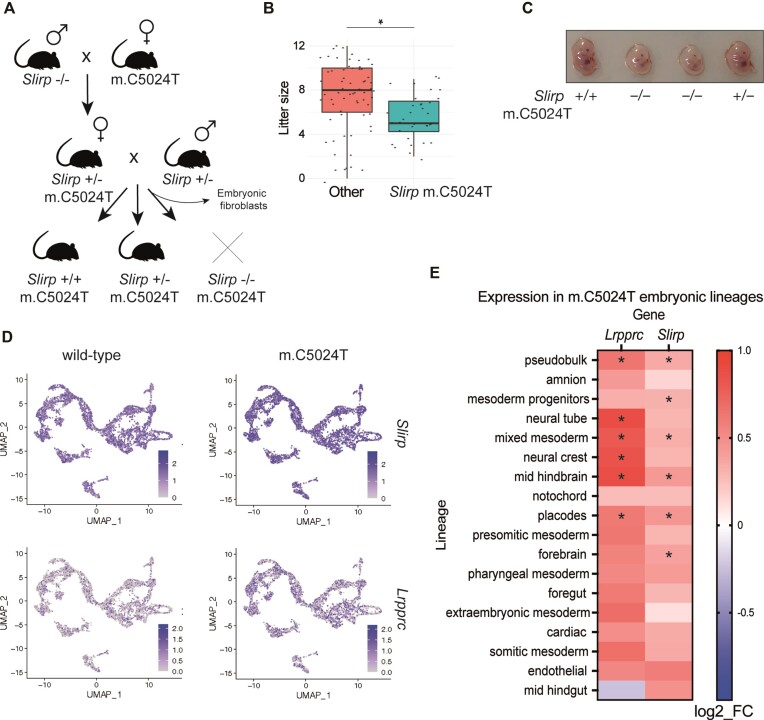
SLIRP is essential in a mouse model carrying the m.C5024T mutation in the tRNA^Ala^ gene of mtDNA. (**A**) Breeding strategy to obtain *Slirp*^−/−^ offspring harboring the m.C5024T mutation in the tRNA^Ala^ gene (*Slirp* -/- m.C5024T). (**B**) Box plot showing the distribution of litter sizes of the *Slirp* m.C5024T breeding line (*Slirp*^+/-^ m.C5024T females mated to *Slirp*^+/−^ males) compared to the litter size distribution of other mouse lines bred in the same facility. *P = 0.004. (**C**) Embryos at E13.5 showing the developmental defect resulting in embryonic lethality of *Slirp*^−/−^ mice harboring the m.C5024T mutation in the mt-tRNA^Ala^ gene (Slirp -/- m.C5024T). (**D**) Uniform Manifold Approximation and Projection (UMAP) showing 17 different cell lineages (defined by known marker-gene expression levels in (34) from wildtype and m.C5024T embryos. Each dot represents a single cell, colour intensity is scaled to expression level of *Lrpprc* or *Slirp* transcripts. (**E**) Heat map of the differential expression of *Lrpprc* and *Slirp* genes in cell lineages from m.C5024T embryos compared with wildtype embryos at E8.5. Stars indicate statistically significant differential expression (adjusted *P*-value < 0.05).

The lack of SLIRP and the concomitant decrease in LRPPRC leads to drastically decreased levels of mt-mRNAs, but the remaining mitochondrial translation capacity is sufficient to sustain normal physiology. Extensive phenotyping of *Slirp*^−/−^ mice has only revealed subtle aberrations such as a reduction of weight gain and a minor increase of blood lactate levels (14). To investigate whether an independent defect in the mitochondrial translation system will be aggravated by lack of SLIRP, we crossed *Slirp*^−/−^ mice to mice carrying the m.C5024T mutation in the tRNA*^Ala^* gene of mtDNA. Mice harboring this heteroplasmic m.C5024T mutation have been extensively characterized and show a slight reduction in weight gain and a moderate increase of heart mass but appear healthy (25). We crossed *Slirp*^+/−^ males with *Slirp*^+/−^ females carrying various levels of the heteroplasmic m.C5024T mutation (54–78%) and obtained *Slirp*^+/+^ m.C5024T and *Slirp*^+/−^ m.C5024T offspring, but no *Slirp*^−/−^ m.C5024T mice (Figure 4A). Characterization of *Slirp*^+/−^ m.C5024T mice did not show significant differences compared to controls (data not shown). The number of pups per litter when *Slirp^+/-^*and *Slirp^+/−^* m.C5024T mice were crossed was significantly smaller in comparison with other lines (Figure 4B, Ext. Figure 4A), consistent with embryonic lethality, and we therefore proceeded with dissection of staged embryos. The *Slirp*^−/−^ m.C5024T embryos survived well past implantation but at E13.5 they were smaller and showed developmental defects (Figure 4C, Ext. Figure 4B, C). To gain a better molecular insight into tissue-specific mechanisms during development, we re-analyzed data from single-cell RNA-seq of embryonic cell linages in m.C5024T mouse embryos at day E8.5 (34). Published results argue that induction of protective responses during embryogenesis explains why the m.C5024T mice have a rather mild phenotype. We reanalyzed these data for *Lrpprc* and *Slirp* transcript levels in single cells from different lineages in E8.5 embryos and found a significant upregulation of both transcripts in m.C5024T embryos in comparison with wildtypes, especially in brain-related lineages (Figure 4D, E, Ext. Figure 4D, E). This finding argues that brain development is particularly vulnerable in the *Slirp*^−/−^ m.C5024T genetic background.

**Figure 5.**
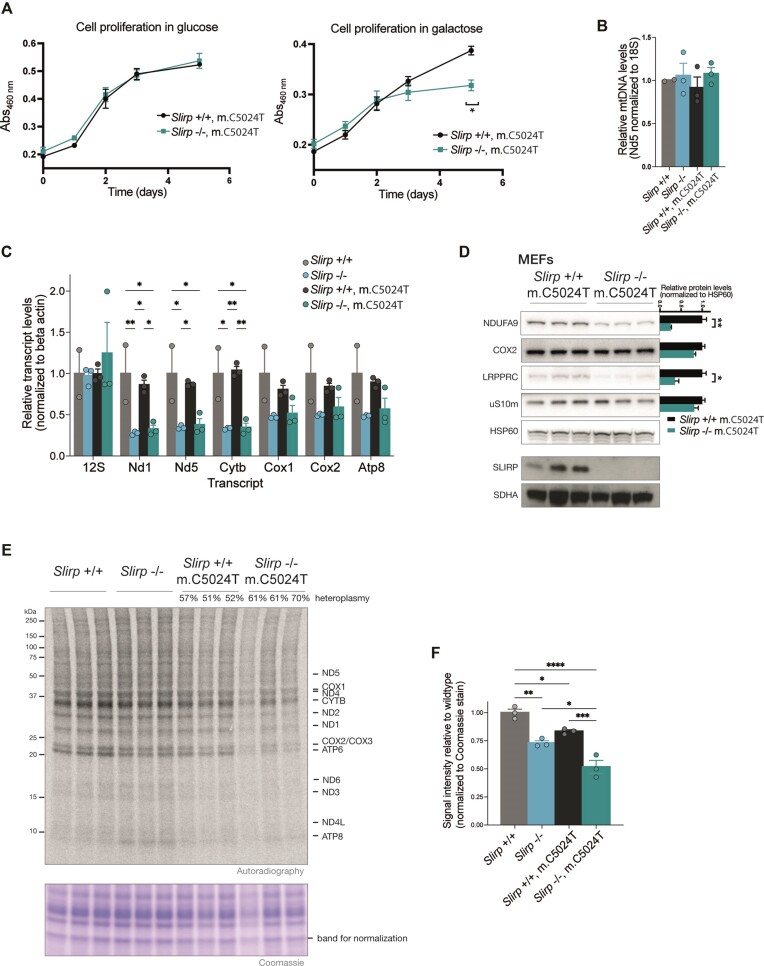
Molecular defects of *Slirp*^−/−^ and the m.C5024T mutation in mouse embryonic fibroblasts. (**A**) Cell proliferation curves of mouse embryonic fibroblasts (MEFs) derived from *Slirp*^+/+^ m.C5024T and *Slirp* knockout^−/−^ m.C5024T embryos. Primary MEFs were grown in media containing glucose (left plot) or galactose (right plot) and their growth was measured by CCK-8 (WST-8) cell viability assays. Error bars are ± SEM, *n* = 3 biological replicates, * adj. *P* value = 0.04 by two-way ANOVA Sidak's multiple comparisons test. (**B**) Quantification of mtDNA levels by qPCR (Nd5/18S genes) in primary MEFs from *Slirp*^+/+^ and *Slirp*^−/−^ embryos harboring wildtype or m.C5024T mtDNA. Bars represent means ± SEM, *n* = 2–3, no significant differences observed by one-way ANOVA Tukey's multiple comparisons test. (**C**) Quantification of mitochondrial transcript levels by RT-qPCR in primary MEFs from *Slirp*^+/+^ and *Slirp*^−/−^ embryos harboring wildtype or m.C5024T mtDNA. Data are represented as means ± SEM, *n* = 2–3 biological replicates, ** adj. *P* value < 0.01, * adj. *P* value < 0.05 by two-way ANOVA Tukey's multiple comparisons test. (**D**) Western blots of steady-state levels of NDUFA9, COX2, LRPPRC, uS10m and SLIRP proteins in *Slirp*^+/+^ m.C5024T and *Slirp*^−/−^ m.C5024T primary MEFs (30 ug). HSP60 was used as a loading control. Densitometric quantifications are shown, error bars are SEM, *n* = 3 biological replicates. * *P* value < 0.05, ** *P* value < 0.005. (**E**) Mitochondrial translation rate assessed by [^35^S]-methionine/cysteine labelling in primary MEFs from *Slirp*^+/+^ and *Slirp*^−/−^ embryos harboring wildtype or m.C5024T mtDNA, the heteroplasmy levels of the cells are indicated. Putative mitochondrial proteins are labelled, and molecular weights are shown to the left of the gel. The Coomassie staining was used a loading control, *n* = 3 biological replicates. (**F**) Densitometric analysis of the autoradiography shown in (E). Bands labelled COX1, ND4, CYTB, ND2, ND1, COX2/COX3 and ATP6 were considered, one band from the Coomassie staining was used to normalize quantities. Data are represented as means ± SEM, *n* = 3 biological replicates, **** adj. *P* value < 0.0001, *** adj. *P* value < 0.001 ** adj. *P* value < 0.01, * adj. *P* value < 0.05 by one-way ANOVA Tukey's multiple comparisons test.

Next, we prepared primary mouse embryonic fibroblasts (MEFs) at day E13.5 and found that *Slirp*^−/−^ m.C5024T MEFs proliferated at a lower rate in galactose media than *Slirp*^+/+^ m.C5024T MEFs (Figure 5A), consistent with an OXPHOS defect. The mtDNA levels were not affected whereas mt-mRNA levels were decreased in *Slirp*^−/−^ m.C5024T MEFs (Figure 5B, C), similar to the findings in *Slirp*^−/−^ MEFs not carrying the m.C5024T mutation (14). Western blots of mitochondrial proteins showed a decrease in the complex I subunit NDUFA9 but not the complex IV subunit COX2 (Figure 5D), in line with a complex I-specific defect observed in *Slirp*^−/−^ mice (Figure 1A, B). We assessed mitochondrial translation in cultured MEFs by radiolabeling newly synthesized proteins with ^35^S-methionine/cysteine in the presence of an inhibitor of cytosolic translation (Figure 5E, Ext. Figure 5A, B). Compared to *Slirp*^+/+^ MEFs, we found ∼30% decrease of mitochondrial translation in *Slirp*^−/−^ MEFs and ∼20% decrease in *Slirp*^+/+^ m.C5024T MEFs. Interestingly, there was an additive effect in *Slirp*^−/−^ m.C5024T MEFs with a decrease of ∼50% in mitochondrial translation (Figure 5F).

Taken together, we report that loss of SLIRP is tolerated in mice unless an additional translation defect is introduced. SLIRP becomes essential when combined with the m.C5024T mutation in the tRNA^Ala^ gene, and without SLIRP the translation defect caused by this mtDNA mutation becomes aggravated. Our results show that genetic interactions between different components of the mitochondrial translation machinery can aggravate phenotypes and that translation levels below 50–70% are incompatible with normal embryonic development, especially of brain lineages.

## Discussion

Our understanding of the coordination of mammalian mtDNA gene expression has expanded considerably in the previous decades. Current research efforts are focused on identifying novel factors, unraveling control mechanisms, and mutating mtDNA with base editing approaches to understand regulation of mtDNA gene expression. Here, we explored the molecular phenotypes caused by mutating LRPPRC and SLIRP *in vivo*, which under normal conditions form a complex to sustain mt-RNA processing and stability, transcript loading, and translation. We combined the reach of proteomics, mouse genetics, transcriptomics, and biochemistry to uncover tissue-specific defects caused by impaired LRPPRC and SLIRP levels. We identified *in vivo* roles for these proteins in maintaining sufficient complex I levels, orderly translation of the *Atp8/Atp6* bicistron, and mouse embryonic development.

We found a decrease in complex I expression in the absence of SLIRP when LRPPRC is decreased to 25% of normal levels. Complex I expression is maintained in *Lrpprc*^+/−^ heart mitochondria when LRPPRC is reduced to 50% and SLIRP to 25% of normal levels, showing that adequate synthesis of complex I subunits is preserved until LRPPRC and SLIRP are profoundly depleted. Our results propose that the expression of complex I is particularly sensitive to a reduction in mtDNA expression. Independent support for this hypothesis comes from studies showing that mt-mRNA turnover and translation efficiency are major predictors of mitochondrial protein synthesis levels in cultured cells where mt-mRNAs that encode complex I subunits exhibit the lowest average stability and translation efficiency (6,38). In addition, the high turnover of the N and Q modules of complex I compared to other OXPHOS complexes (39,40) may explain why we observe a decrease in complex I protein levels although mitochondrial translation in heart is only moderately affected. Furthermore, other mouse models with defective mitochondrial gene expression, such as the *Mtif3* and *Grsf1* knockouts, also exhibit a decrease in proteins specific to complex I (41,42).

In the differential proteomics analysis of immunoprecipitated ribosomes reported here, the only detected factors that clustered in their function of mitoribosome biogenesis and translation were MTIF3, GFM2, and MALSU1, which were detected in liver but not in heart. These enrichments did not reach the FDR cut-off and require additional investigation. However, we previously reported that the lack of SLIRP in liver results in reduced mitochondrial translation which may be accompanied by a compensatory increase in mitoribosome biogenesis.

The *Slirp*^−/−^ model exhibits a clear defect in mitochondrial translation in liver and kidney, and no marked changes in heart (14), whereas mt-mRNAs are decreased in all these tissues. Differential proteomics analysis of immunoprecipitated ribosomes and ribosomal profiling of mt-mRNA loading support that mitochondrial translation is compromised in liver as opposed to the heart. A potential explanation could be that the long half-life of OXPHOS complexes in heart (40) reduces the need for mitochondrial translation and that low levels of mt-mRNAs therefore are sufficient for near-normal translation even in the absence of SLIRP. Alternatively, this tissue-specific effect could be explained by an unknown compensatory mechanism in heart. These results add to the increasing evidence of tissue-specificity in the regulation of mammalian mtDNA gene expression (34,43,44). Our understanding of this phenomenon is relevant in clinical research as mitochondrial diseases have different manifestations depending on the tissue that harbors the mutation (45,46).

In *Lrpprc*^ki/ki^ livers, where the interaction between LRPPRC and SLIRP has been disrupted, the synthesis of ATP8 increased while overall mitochondrial translation is decreased. In comparison, translation of the *Nd4L/Nd4* bicistronic mt-mRNA was reduced to the same extent as the translation of other mt-mRNAs. It remains to be determined if the defect in the synthesis of ATP8 is specific to the amino acid substitutions of LRPPRC introduced in this work. In fibroblasts derived from patients carrying mutations of LRPPRC, the defect in mitochondrial translation varies and often shows a general decrease without any distinct increase in ATP8 production (Oláhová et al., 2015; Sasarman et al., 2010). Also, knockout of *Lrpprc* in mouse heart and skeletal muscle results in uncoordinated mitochondrial translation. Our results show that LRPPRC plays a specific role in determining transcript preference for the synthesis of ATP8 and supports the potential impact of LRPPRC and SLIRP in influencing the secondary structure of the *Atp8/Atp6* bicistronic mt-mRNA (15,47).

We observed sedimentation profiles characteristic of increased mitoribosome polysome (mitopolysomes) formation in *Lrpprc*^ki/ki^ and *Slirp*^−/−^ mitochondria which were much less abundant or not present in wildtype samples. Studies on cultured mammalian cells have reported the existence of mitopolysomes (38,48,49). However, mitopolysomes are typically not seen in normal mammalian tissues, pointing towards fundamental differences between molecular phenotypes of cultured cells and differentiated tissues. The resolution of the sucrose gradients we performed allowed observation of up to three RNA peaks that sedimented after the monosome fractions, whereas other studies on mouse tissues have detected RNA peaks in the last fractions, immediately after the monosome, which may correspond to unresolved mitopolysomes (11,14,41). When SLIRP/LRPPRC levels are low, we hypothesize that the decrease in mt-mRNA abundance together with poor coordination of mitochondrial translation result in the binding of multiple mitoribosomes to single mt-mRNAs causing a polysome-like pattern on sucrose gradients.

Knockout of *Slirp* and the m.C5024T mutation of the tRNA^Ala^ gene of mtDNA affect mitochondrial translation at different steps, but the gross physiology of mice with either genetic defect remains mostly unaffected (14,25). Here, we report that a combination of these two defects leads to a synergistic decrease in protein synthesis below a critical threshold compatible with normal embryonic development. Several mouse models lacking genes involved in mtDNA gene expression display embryonic lethality at E8.5, including homozygous full-body knockouts for *Tfam*, *Tefm*, *Polrmt*, *Polg*, *Twinkle*, *Ssbp1*, *Mrpp3*, *Elac2* and *Lrpprc* (7,11,50–56). In this study, we combined two essentially healthy mouse models and observed that the *Slirp^−/−^*, m.C5024T embryos could develop until stage E13.5. Threshold effects in physiology were introduced almost a century ago (57) and have been revisited with a focus on mitochondrial biology in recent years (58,59). For instance, thresholds in the heteroplasmy levels of mtDNA copies are consistently observed for the manifestation of disease phenotypes (45,60). Another example is the critical TFAM-to-mtDNA ratio in mouse models, where a moderate increase in TFAM levels allows normal physiology while high overexpression leads to early postnatal lethality (43).

Overall, we show that LRPPRC and SLIRP are implicated in several tissue-specific phenotypes such as maintaining complex I levels, coordinating the preferential translation of mt-mRNAs, and enabling mitochondrial protein synthesis rate during embryogenesis. These findings not only add to our understanding of the mechanisms leading to mitochondrial disease phenotypes but also contributes to the growing knowledge of fundamental mitochondrial biology.

## Supplementary Material

gkae662_Supplemental_Files

## Data Availability

The LC-MS/MS data have been deposited to the ProteomeXchange Consortium via the PRIDE (30) partner repository with the dataset identifier PXD046268.
