# Supplementary material for: LRPPRC and SLIRP synergize to maintain sufficient and orderly mammalian mitochondrial translation
Source: Nucleic Acids Res. 2024 Aug 1;52(18):11266–82. doi: 10.1093/nar/gkae662 (PMC11472161; doi:10.1093/nar/gkae662)
Supplement: gkae662_Supplemental_Files [file gkae662_supplemental_files.zip › Supplementary_data 2.pdf]

Supplementary materials for

LRPPRC and SLIRP synergize to maintain sufficient and orderly mammalian mitochondrial translation

Diana Rubalcava-Gracia[#1], Kristina Bubb[1], Fredrik Levander[2], Stephen Burr[3], Amelie V. August[1], Patrick Chinnery[3], Camilla Koolmeister[1], Nils-Göran Larsson[#1]

# Corresponding authors: diana.rubalcava@ki.se, nils-goran.larsson@ki.se

This PDF file includes:

Extended figures 1-5.
Supplementary tables with sequences, reagents, and resources.

Extended Figure 1

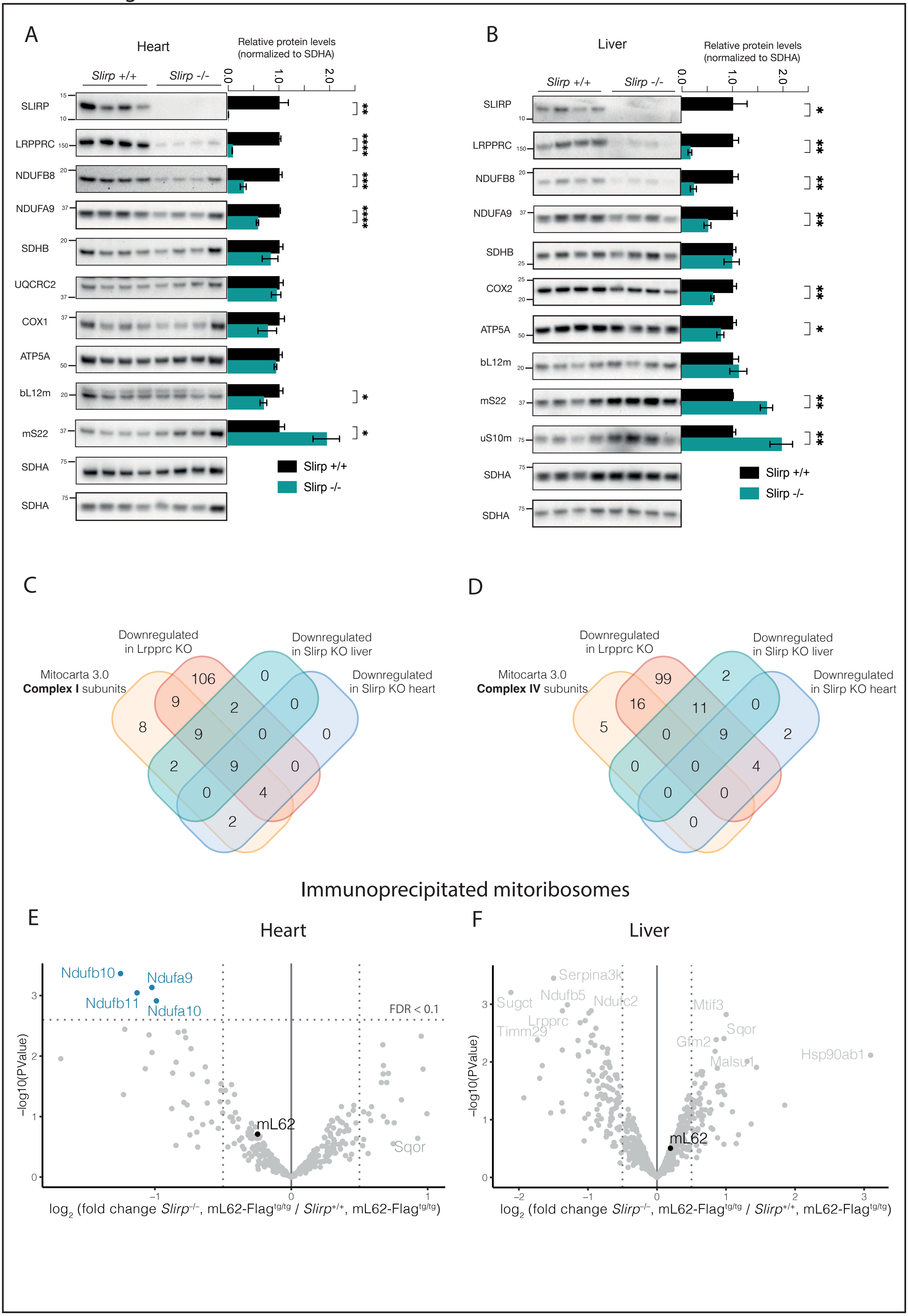

Extended Figure 1. A, B) Western blots of steady-state levels of LRPPRC, SLIRP, and OXPHOS subunits in heart (A) and liver (B) mitochondria (10 ug) from 12-week-old *Slirp*$^{+/+}$ and *Slirp*$^{-/-}$ mice. SDHA was used as a loading control. Densitometric quantification is shown for each blot, error bars are SEM, n=5 biological replicates. * p value < 0.05, ** p value < 0.005, *** p value < 0.0001. **C, D)** Venn diagrams depicting downregulated proteins in *Slirp*$^{-/-}$ tissues in comparison with complex I (C) and IV (D) subunits from Mitocarta 3.0 or the proteomic datasets from conditional *Lrpprc*$^{-/-}$ hearts (from Kühl et al. 2017). **E, F)** Proteomic comparison of immunoprecipitated mitoribsomes in heart (E) and liver (F). Percoll-purified mitochondria of 12-week-old *Slirp*$^{-/-}$, mL62-Flag$^{tg/tg}$ and *Slirp*$^{+/+}$, mL62-Flag$^{tg/tg}$ mice were subjected to anti-FLAG immunoprecipitation and proteomic analysis. The dashed line marks the false discovery rate cut-off (FDR<0.1). Note that in liver, no proteins passed the FDR<0.1 cut-off.

Extended Figure 2

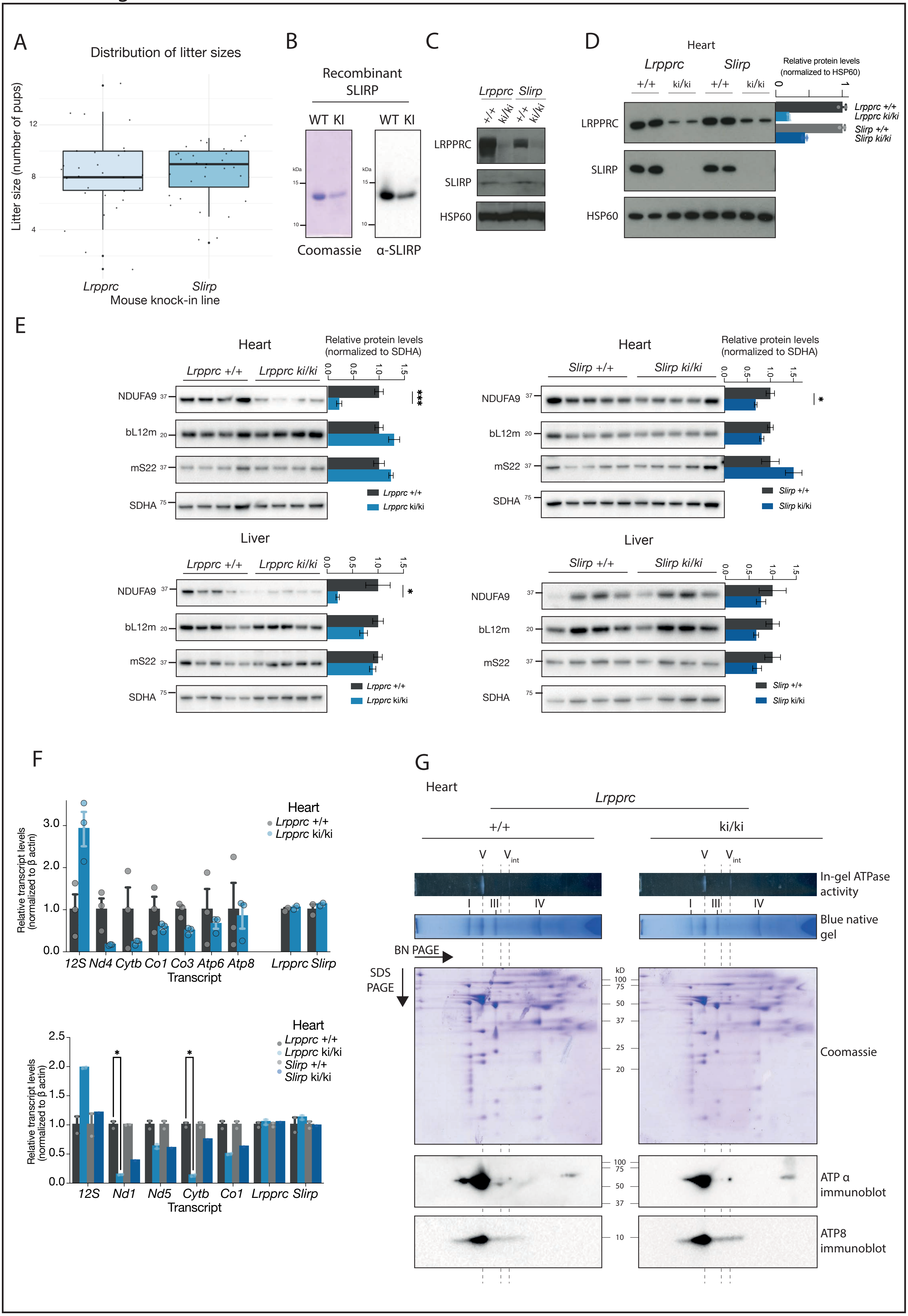

Extended Figure 2. A) Box plot showing the distribution of litter sizes of the *Lrpprc*$^{ki/ki}$ and *Slirp*$^{ki/ki}$ lines. **B)** Western blot of the recombinant wildtype and knock-in versions of SLIRP using the anti-SLIRP antibody. **C)** Western blots of steady-state protein levels of LRPPRC and SLIRP in whole-tissue extracts from *Lrpprc*$^{ki/ki}$ and *Slirp*$^{ki/ki}$ hearts. **D)** Western blots of steady-state protein levels of LRPPRC and SLIRP in heart mitochondria from 8-week-old control (+/+) and *Lrpprc*$^{ki/ki}$ and *Slirp*$^{ki/ki}$ mutants. HSP60 was used as a loading control. **E)** Western blots of steady-state protein levels of NDUFA9 and mitoribosome subunits in heart and liver mitochondria from control (+/+) and *Lrpprc*$^{ki/ki}$ and *Slirp*$^{ki/ki}$ mice. SDHA was used as a loading control. **F)** RT-qPCR analyses of transcript levels in heart mitochondria from control (+/+), *Lrpprc*$^{ki/ki}$, and *Slirp*$^{ki/ki}$ mice. **G)** Second dimension electrophoresis of solubilized mitochondria for immunodetection of ATPα and ATP8. Heart mitochondria from *Lrpprc*$^{+/+}$ and *Lrpprc*$^{ki/ki}$ mice were solubilized with dodecyl-maltoside and resolved by BN-PAGE (first dimension) in duplicate. One duplicate lane was incubated with substrates for ATPase in-gel activity, the other duplicate lane was excised and further resolved by LDS-PAGE (second dimension). The positions of the fully assembled OXPHOS complexes I, III, IV and V, as well as the subassembly intermediate of ATP synthase are indicated as dotted lines. The Coomassie stain and the western blots in the second-dimension gel are aligned to the first-dimension gel.

Extended Figure 3

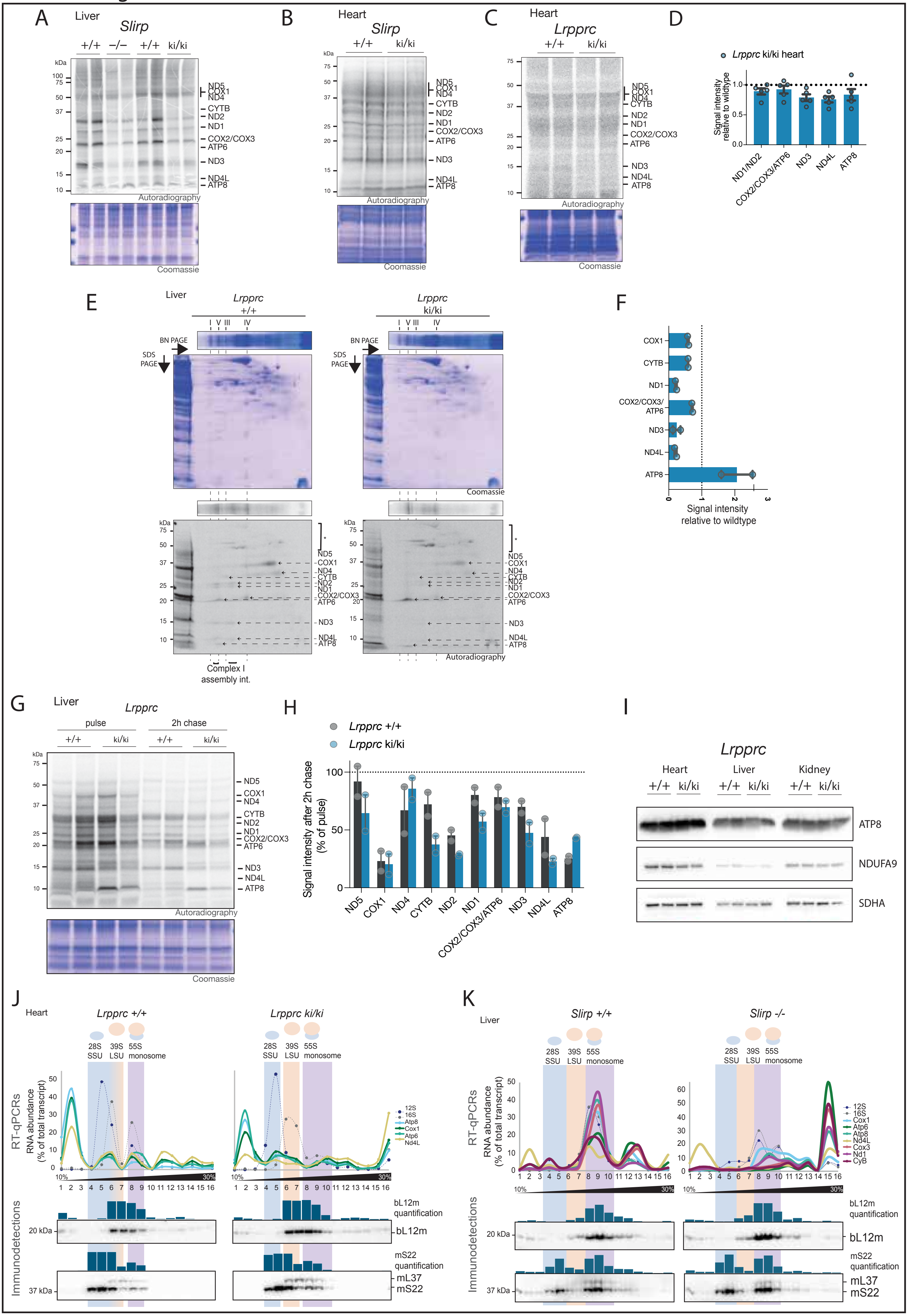

Extended Figure 3. A-C) Mitochondrial translation assessed by *in organello* [^{35}S]-methionine labelling, LDS-PAGE and digital autoradiography in **a)** liver mitochondria from *Slirp*$^{+/+}$, *Slirp*$^{-/-}$ and *Slirp*$^{ki/ki}$ 18-week-old mice, **b)** heart mitochondria from *Slirp*$^{+/+}$ and *Slirp*$^{ki/ki}$ 35-week-old mice, **c)** heart mitochondria from *Lrpprc*$^{+/+}$ and *Lrpprc*$^{ki/ki}$ 35-week-old mice. Coomassie staining was used as a loading control. **D)** Quantification of signal intensity relative to wildtype control of mitochondrial translation products in *Lrpprc*$^{ki/ki}$ mice as shown in Ext. Fig. 3C. n=5 biological replicates. **E)** Extended images of second-dimension electrophoresis of mitochondrial translation products (Fig. 3C). Newly synthesized proteins were labelled for 1h in the presence of [^{35}S]-methionine followed by a 40-minute chase in liver mitochondria isolated from control (+/+) and *Lrpprc*$^{ki/ki}$ 14-week-old mice. Mitochondria were solubilized with dodecyl-maltoside and resolved by BN-PAGE (first dimension). Each lane was excised and further resolved by LDS-PAGE (second dimension). The first well of the second-dimension gel was loaded with mitochondria (75 µg) in LDS sample buffer. Gels were stained with Coomasie (upper panels) and submitted to digital autoradiography (lower panels). The positions of the fully assembled OXPHOS complexes I, III, IV and V are indicated in the first-dimension gel and mtDNA-encoded subunits are indicated in the second-dimension gel. The proposed position of a Complex I assembly intermediate is labelled. The asterisk (*) indicates [^{35}S]-labelled products that were incompletely denatured. **F)** Signal intensity quantification relative to wildtype control of mitochondrial [^{35}S]-labelled translation products shown in Fig. 3C and Ext. Fig. 3E. **G)** Mitochondrial protein synthesis and turnover rates assessed by *in organello* [^{35}S]-methionine labelling of liver mitochondria from *Lrpprc*$^{+/+}$ and *Lrpprc*$^{ki/ki}$ mice. Newly synthesized proteins were labelled for 1h with [^{35}S]-methionine followed by a 2-hour chase in the presence of cold methionine. Coomassie staining was used as a loading control. **H)** Signal intensity quantification of mitochondrial translation products relative to [^{35}S] pulse and after a 2-hour chase in *Lrpprc*$^{+/+}$ and *Lrpprc*$^{ki/ki}$ samples shown in Ext. Fig. 3G. **I)** Western blots of steady-state levels of ATP8 and NDUFA9 in heart, liver and kidney mitochondria (15 µg) from *Lrpprc*$^{+/+}$ and *Lrpprc*$^{ki/ki}$ mice. SDHA was used as a loading control. **J)** Sedimentation profiles in sucrose gradients (10-30%) for individual mt-transcripts detected by RT-qPCR (upper panel) in heart mitochondria isolated from *Lrpprc*$^{+/+}$ and *Lrpprc*$^{ki/ki}$ mice. The plotted percentage for each transcript is normalized to the total quantity for that transcript across all 16 fractions. Immunodetections and quantification of the indicated mitoribosome proteins are shown in the bottom panels. **K)** Sedimentation profiles in sucrose gradients (10-30%) for individual mt-transcripts detected by RT-qPCR (upper panel) in liver mitochondria isolated from *Slirp*$^{+/+}$ and *Slirp*$^{-/-}$ mice. The plotted percentage for each transcript is normalized to the total quantity for that transcript across all 16 fractions. Immunodetections and quantification of the indicated mitoribosome proteins are shown in the bottom panels.

Extended Figure 4

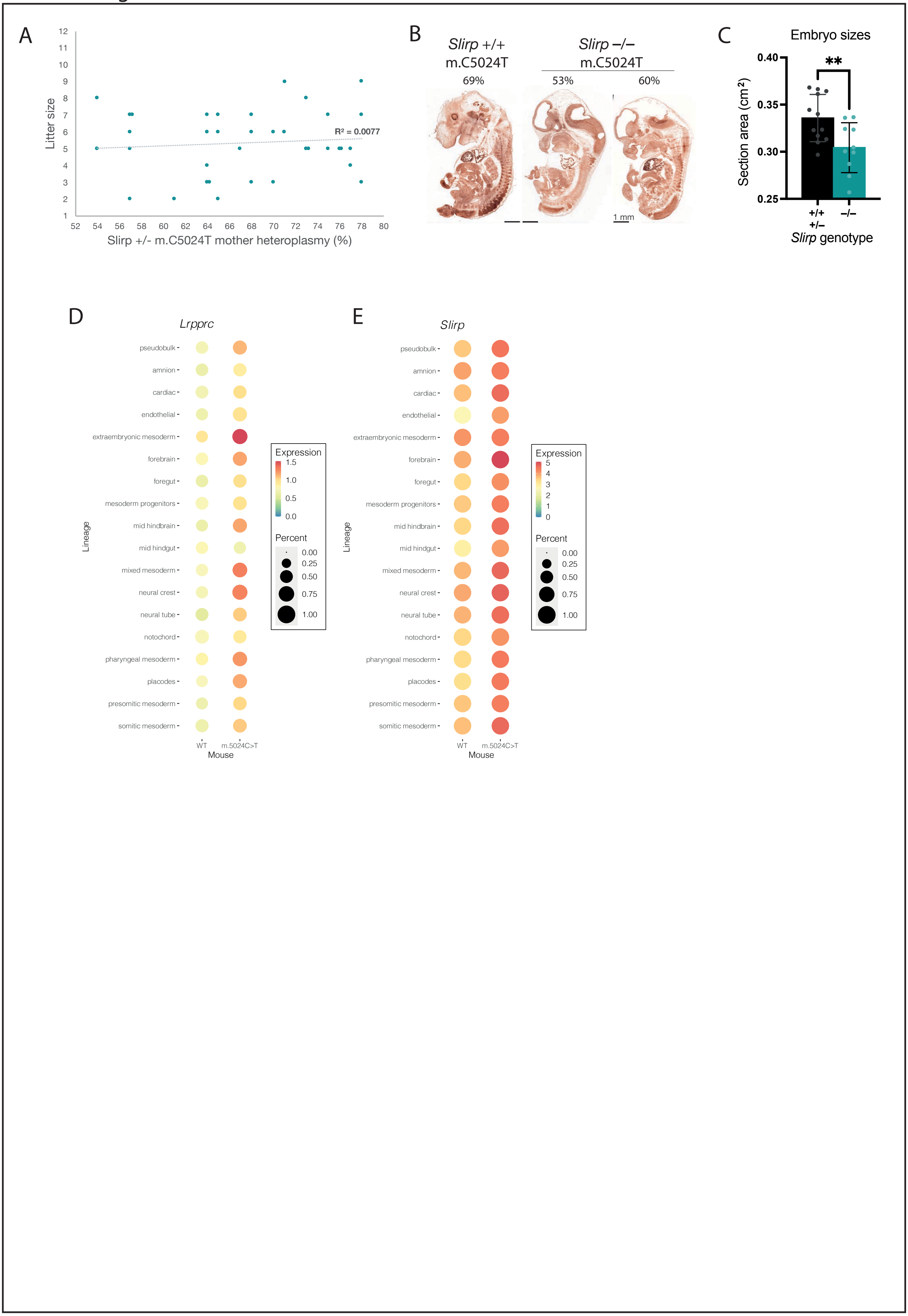

Extended Figure 4. A) Scatterplot of litter sizes as a function of the heteroplasmy level of the mother (ear biopsy) for the *Slirp*$^{-/-}$ m.C5024T line. Trendline and R^2 value are shown.
B) COX/SDH staining of OCT-embedded embryos at stage E13.5 from *Slirp*$^{+/+}$ m.C5024T and Slirp$^{-/-}$ m.C5024T mice. The heteroplasmy level of each embryo is indicated. Scale bars are 1 mm. **C)** Quantification of the total area (cm^2) of the sagittal plane sections of control (*Slirp*$^{+/+}$ m.C5024T and *Slirp*$^{+/-}$ m.C5024T) and *Slirp*$^{-/-}$ m.C5024T embryos represented in Ext. Fig. 4B. **D, E)** Dot plots showing the relative expression of d) *Lrpprc* and *e) Slirp* genes in embryonic cell lineages of wildtype and m.C5024T embryos at E8.5. The size of each dot is proportional to the fraction of cells within that lineage that had detectable expression of *Lrpprc* or *Slirp* genes. The colour of the dot is scaled to the expression level.

Extended Figure 5

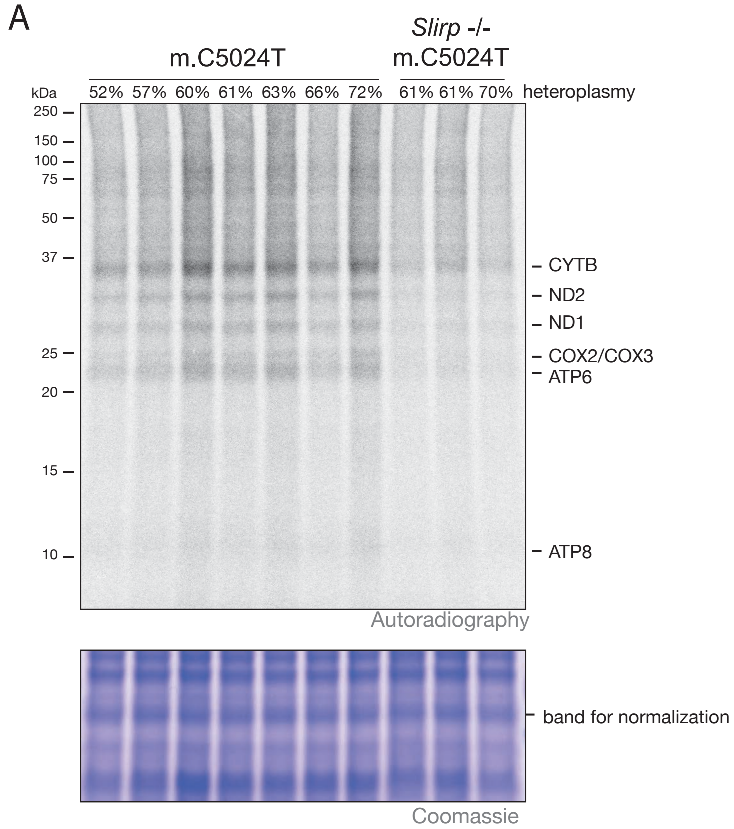

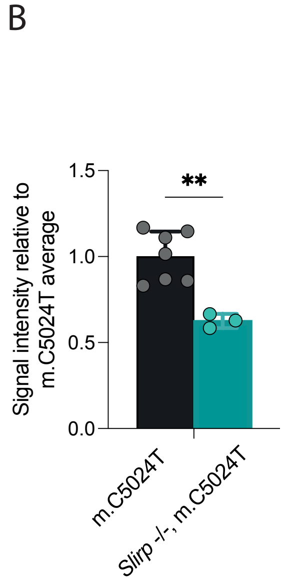

Extended Figure 5. A) Mitochondrial translation rates assessed by [^{35}S]-methionine/cysteine labelling in primary MEFs harboring the m.C5024T mutation in mtDNA compared to MEFs from *Slirp*$^{-/-}$ embryos also harboring the m.C5024T mutation. Heteroplasmy levels are indicated, putative mitochondrial proteins are labelled, and molecular weights are shown to the left autoradiography. Coomassie staining was used a loading control, n = 3-7 biological replicates. **B)** Signal intensity quantification of mitochondrial translation products shown in Ext. Fig. 5A. Bands labelled CYTB, ND2, ND1, COX2/COX3 and ATP6 were considered, one band from the Coomassie staining was used to normalize quantities. Data are represented as means ± SD, n=3-7 biological replicates, ** adj. p value < 0.01 by unpaired t test.

Table S3. Sequences, reagents, and resources

sgRNA

Target gene	Sequence
Lrpprc	CAGAAATAGTGCGGTCGGAT
Slirp	CAAGAACATCATATTATTGA

ssODN (EarI restriction site created)

Target gene	Sequence
Lrpprc	AAACACTCTGACTAGTTCTGACCCCATAAAAGGCAACC<br>TCACCTTGAACATTTTTTGTTTTCTGATGCCCAGCAAG<br>aAgaGGCCAGAAAgcGgcCGGTgcGATAGGAAACCCTTC<br>TTCCCTCAAAGCCTCCATCACTGCTTTTGCCAAAGCTG<br>AAAAAAACAGAAATAGAAATCACAGACCACGTCACCAG<br>GCTAGCTCA
Slirp	TTTAGGACAAAGAGACTGGCTTTCACAGAGGCATGGG<br>TTGGGTTCAGTTTTCCTCTCAGGAAGAACTTCAGAATG<br>CtCTtCAACAAGcAgcTgcTATTATTGATGGAGTAAAAGTA<br>TTTCCGATGTGTGCTCCACGTCCCTTTAAGAGTAGTTA<br>TGTCGTGCGTGCGAGTCTGAAAAGGGAAGTCCCTGAA<br>GACCT

Primary antibodies

Target	Source	Cat. No.
LRPPRC	Sigma-Aldrich	SAB-2700419
SLIRP	Santa Cruz	sc-514508
OXPHOS cocktail	abcam	ab110413
SDHA	abcam	ab14715
HSP60	Enzo Life Sciences	ADI-SPA-807-E
NDUFA9	abcam	ab14713
COX2 (MTCO2)	homemade antisera	
MRPL12	Millipore Sigma	HPA022853
MRPL37	Merck	HPA025826
MRPS22	Thermo Fisher	10984-1-AP
MRPS10	Novus	NBP1-83848
GRSF1	sigma	AV40382
TACO1	ProteinTech	21147-1-AP
MTIF3	ProteinTech	14219-1-AP
MTIF2	Abcam	AB197771
FASTKD2	Proteintech	17464-1-AP
ATP5A (ATP α)	Abcam	ab14748
ATP8	homemade antisera	

Secondary antibodies		
Anti-rabbit	Cytiva	NA9340V
Anti-mouse	Cytiva	NA9310V

qPCR Taqman probes

Target	Source	Identifier	Notes
12S	Thermo Fisher Scientific	Mm04260177_s1	
16S	Thermo Fisher Scientific	Mm04260181_s1	
18S	Thermo Fisher Scientific	Mm03928990_g1	
Actin	Thermo Fisher Scientific	Mm01205647_g1	
Atp6	Thermo Fisher Scientific	Mm03649417_g1	
Atp8	Thermo Fisher Scientific	Mm04225236_g1	
Cox1	Thermo Fisher Scientific	Mm04225243_g1	
Cox2	Thermo Fisher Scientific	Mm03294838_g1	
Cox3	Thermo Fisher Scientific	Mm04225261_g1	
Cyb	Thermo Fisher Scientific	Mm04225271_g1	
Nd1	Thermo Fisher Scientific	Mm04225274_g1	
Nd4l/4	Thermo Fisher Scientific	Mm04225294_s1	
Nd5	Thermo Fisher Scientific	Mm04225315_s1	Also recognizes *Nd6*
Slirp	Thermo Fisher Scientific	Mm01296845_m1	
Lrpprc	Thermo Fisher Scientific	Mm00511512_m1	

Templates used to prepare Northern blot probes for mouse

(cloned into PCRII TOPO vector, released by EcoRI digestion)

Target	Sequence
Cox1	GTTCATTATTTTTGGTTGGTTGTCTTGGGTTAGCATTAAAGCCTTCACCTATTTA<br>TGGAGGTTTAGGTTTAATTGTTAGTGGGTTTGTTGGTTGTTTAATGGTTTTAGGG<br>TTTGGTGGATCGTTTTTAGGTTTAATAGTTTTTTAATTTATTTAGGGGGGATGT<br>TGGTTGTGTTTGGATATACGACTGCTATAGCTACTGAGGAATATCCAGAGACTT<br>GGGGATCTAACTGATTAATTTTGGGTTTTTTAGTATTGGGGGTGATTATAGAGG<br>TTTTTTTAATTTGTGTGCTTAATTATTATGATGAAGTTGGAGTAATTAATCTTGA<br>TGGTTTGGGAGATTGGTTGATGTATGAGGTTGATGATGTTGGAGTTATGTTGGA<br>AGGAGGGATTGGGGTAGCGGCAATATATAGTTGTGCTACTTGAATGATGGTAG<br>TAGCTGGGTGATCTTTGTTTGCGGGTATTTTTATT
Cox2	GGTCTACAAGACGCCACATCCCCTATTATAGAAGAGCTAATAAATTTCCATGAT<br>CACACACTAATAATTGTTTTCCTAATTAGCTCCTTAGTCCTCTATATCATCTCGC<br>TAATATTAACAACAAAACTAACACATACAAGCACAATAGATGCACAAGAAGTT<br>GAAACCATTTGAACTATTCTACCAGCTGTAATCCTTATCATAATTGCTCTCCCCT<br>CTCTACGCATTCTATATATAATAGACGAAATCAACAACCCCGTATTAACCGTTA<br>AAACCATAGGGCACCAATGATACTGAAGCTACGAATATACTGACTATGAAGAC<br>CTATGCTTTGATTCATATATAATCCCAACAAACGACCTAAAACCTGGTGAACTA<br>CGACTGCTAGAAGTTGATAACCGAGTCGTTCTGCCAA
Cox3	ND
CytB	AGTAGACAAAGCCACCTTGACCCGATTCTTCGCTTTCCACTTCATCTTACCATTT<br>ATTATCGCGGCCCTAGCAATCGTTCACCTCCTCTTCCTCCACGAAACAGGATCA<br>AACAACCCAACAGGATTAAACTCAGATGCAGATAAAATTCCATTTCACCCCTA<br>CTATACAATCAAAGATATCCTAGGTATCCTAATCATATTCTTAATTCTCATAAC<br>CCTAGTATTATTTTTCCCAGACATACTAGGAGACCCAGACAACTACATACCAGC<br>TAATCCACTAAACACCCCACCCCATATTAAACCCGAATGATATTTCCTATTTGC<br>ATACGCCATTCTACGCTCAATCCCCAATAAACTAGGAGGTGTCCTAGCCTTAAT<br>CTTATCTATCCTAATTTTAGCCCTAATACCTTTCCTTCATACCTCAAAGCAACGA<br>AGCCTAATATTCCGCCCAATCACACAAATTTTGTACTGAATCCTAGTAGCCAAC<br>CTACTTATCTTAACCTGAATTGGGGGCCAACCAGTAGAACACCCATTT
Nd5	ACAAGACATCCGAAAAATAGGAAACATCACAAAAATCATACCATTCACATCAT<br>CATGCCTAGTAATCGGAAGCCTCGCCCTCACAGGAATACCATTCCTAACAGGG<br>TTCTACTCAAAAGACCTAATTATTGAAGCAATTAATACCTGCAACACCAACGCC<br>TGAGCCCTACTAATTACACTAATCGCCACTTCTATAACAGCTATGTACAGCATA<br>CGAATCATTTACTTCGTAACAATAACAAAACCGCGTTTTCCCCCCCTAATCTCC<br>ATTAACGAAAATGACCCAGACCTCATAAACCCAATCAAACGCCTAGCATTCGG<br>AAGCATCTTTGCAGGATTTGTCATCTCATATAATATTCCACCAACCAGCATTCC<br>AGTCCTCACAATACCATGATTTTTAAAAACCACAGCCCTAATTATTTCAGTATT<br>AGGATTCCTAATCGCACTAGAACTAAACAACCTAACCATAAAACTATCAATAA<br>ATAAAGCAAATCCATATTCATCCTTCTCAACTTTACTGGGGTTTTTCCCATCTAT<br>TATTCACCGCATTACACC
12S	TACACATGCAAACCTCCATAGACCGGTGTAAAATCCCTTAAACATTTACTTAAA<br>ATTTAAGGAGAGGGTATCAAGCACATTAAAATAGCTTAAGACACCTTGCCTAG<br>CCACACCCCCACGGGACTCAGCAGTGATAAATATTAAGCAATAAACGAAAGTT<br>TGACTAAGTTATACCTCTTAGGGTTGGTAAATTTCGTGCCAGCCACCGCGGTCA<br>TACGATTAACCCAAACTAATTATCTTCGGCGTAAAACGTGTCAACTATAAATAA<br>ATAAATAGAATTAAAATCCAACTTATATGTGAAAATTCATTGTTAGGACCTAA<br>ACTCAATAACGAAAGTAATTCTAGTCATTTATAATACACGACAGCTAAGACCC<br>AAACTGGGATTAGATACCCCACTATGCTTAGCCATAAACCTAAATAATTAAATT<br>TAACAAAACTATTTGCCAGAGAACTACTAGCCATAGCTTAAAACTCAAAGGAC<br>TTGGCGGTACTTTATATCCATCTAGAGGAGCCTGTTCTATAATCGATAAACCCC<br>GCTCTACCTCACCATCTCTTGCTAA
16S	ND
18S	GGTCTACAAGACGCCACATCCCCTATTATAGAAGAGCTAATAAATTTCCATGAT<br>CACACACTAATAATTGTTTTCCTAATTAGCTCCTTAGTCCTCTATATCATCTCGC<br>TAATATTAACAACAAAACTAACACATACAAGCACAATAGATGCACAAGAAGTT<br>GAAACCATTTGAACTATTCTACCAGCTGTAATCCTTATCATAATTGCTCTCCCCT<br>CTCTACGCATTCTATATATAATAGACGAAATCAACAACCCCGTATTAACCGTTA<br>AAACCATAGGGCACCAATGATACTGAAGCTACGAATATACTGACTATGAAGAC<br>CTATGCTTTGATTCATATATAATCCCAACAAACGACCTAAAACCTGGTGAACTA<br>CGACTGCTAGAAGTTGATAACCGAGTCGTTCTGCCAA

3' RACE assay oligos

Target	Sequence	Amplicon size
Linker	[Phos]-ATGTGAGATCATGCACAGTCATA-[3dA]	NA
Anti-linker	GACTGTGCATGATCTCAC	NA
Nd1	GGATCCGAGCATCTTATCCA	160-210 bp
Nd4	AGTCCAAAACTAATTACAGGCCT	95-110 bp
Nd6	ATTAGTAGGGCTCAGGCGTT	70 bp
Cytb	ACCAATCTCAGGAATTATCGAAG	60-130 bp
Cox1	ACACATTCGAGGAACCAACC	140-200 bp
Cox2	AGGCCGACTAAATCAAGCAA	180-240 bp
Atp8/6	ACCAATGGCATTAGCAGTCC	250-300 bp
